# A Rhein-Based Derivative Targets *Staphylococcus aureus*

**DOI:** 10.3390/antibiotics13090882

**Published:** 2024-09-13

**Authors:** Xiaojia Liu, Yuan Liu, Meirong Song, Kui Zhu, Jianzhong Shen

**Affiliations:** National Key Laboratory of Veterinary Public Health and Safety, College of Veterinary Medicine, China Agricultural University, No. 2 Yuanmingyuan West Road, Beijing 100193, China; liuxj@cau.edu.cn (X.L.); sy20233051144@cau.edu.cn (Y.L.); meirong_song@cau.edu.cn (M.S.)

**Keywords:** rhein, anthraquinone derivatives, Gram-positive pathogen, membrane target

## Abstract

The rise in antibiotic-resistant bacteria highlights the need for novel antimicrobial agents. This study presents the design and synthesis of a series of rhein (RH)-derived compounds with improved antimicrobial properties. The lead compound, RH**17**, exhibited a potent antibacterial activity against *Staphylococcus aureus* (*S. aureus*) isolates, with minimum inhibitory concentrations (MICs) ranging from 8 to 16 μg/mL. RH**17** disrupted bacterial membrane stability, hindered metabolic processes, and led to an increase in reactive oxygen species (ROS) production. These mechanisms were confirmed through bacterial growth inhibition assays, membrane function assessments, and ROS detection. Notably, RH**17** outperformed the parent compound RH and demonstrated bactericidal effects in *S. aureus*. The findings suggest that RH**17** is a promising candidate for further development as an antimicrobial agent against Gram-positive pathogens, addressing the urgent need for new therapies.

## 1. Introduction

The growing issue of antimicrobial resistance in bacteria poses a significant global threat, as it diminishes the effectiveness of current treatments and limits options for managing certain persistent infections [1,2,3]. Methicillin-resistant *Staphylococcus aureus* (MRSA) significantly contributes to mortality among antibiotic-resistant pathogens [4,5,6,7]. MRSA infections are complicated to treat due to their ability to form biofilms and exist as metabolically inactive persister cells, which are both highly tolerant to antibiotics [8,9,10]. Therefore, the development of new therapeutic strategies that effectively target both biofilm-forming bacteria and persisters is crucial. Ideally, new antibacterial agents should disrupt biofilm integrity and demonstrate potent activity against actively growing bacteria and persisters [11,12].

With their structural diversity and biological activity, natural products are a valuable source for drug discovery [13,14,15]. Rhein (RH), an anthraquinone derived from rhubarb (*Rheum palmatum*) [16], has attracted attention due to its wide range of biological activities, including anti-inflammatory [17,18], anticancer [19,20], antiviral [21,22], and antimicrobial properties [23,24,25]. Although RH has demonstrated some antimicrobial activity, with a minimum inhibitory concentration (MIC) of 64 μg/mL against *S. aureus* ATCC 25923, its therapeutic potential is limited due to its relatively low potency [26]. In recent years, various studies have modified the chemical structure of RH to enhance its antibacterial efficacy, achieving some success [27,28].

Cationic antimicrobial peptides (CAMPs) are another promising class of antimicrobial agents known for their potent broad-spectrum activity [29,30]. CAMPs function by interacting with bacterial membranes, thereby causing cell lysis and bacterial death due to their amphiphilic structure, which consists of positively charged hydrophilic regions and hydrophobic tails [31,32]. This unique mechanism of action makes CAMPs particularly effective against drug-resistant pathogens. However, the clinical application of CAMPs has been limited due to challenges with toxicity and stability [33,34,35]. To overcome these limitations, researchers have developed small-molecule mimics of CAMPs, which retain their membrane-disrupting capabilities while minimizing toxicity [32]. Some CAMP mimetics have shown promise in clinical trials, such as LTX-109 (phase Ⅱ) [36] and PMX30063 (phase Ⅱ) [37], demonstrating solid antibacterial activity while reducing the likelihood of bacterial resistance. These small-molecule mimics effectively disrupt bacterial membranes, providing a potent antimicrobial option with improved safety profiles.

In this study, we aimed to develop novel rhein derivatives by incorporating features of CAMPs, such as amphiphilicity and a membrane-targeting ability, to enhance their antibacterial activity. When compared to the parent RH compound, our lead compound, RH**17**, exhibited a significantly improved activity against *S. aureus*, with an MIC of 8 μg/mL. Additionally, RH**17** demonstrated a dual mechanism of action, disrupting bacterial membrane homeostasis and collapsing bacterial metabolism. This study suggests that RH**17** and its analogs represent a new class of membrane-targeting antibacterial agents with the potential for further development.

## 2. Results

### 2.1. Rational Design and Synthesis of RH Analogs

**Figure 1:**
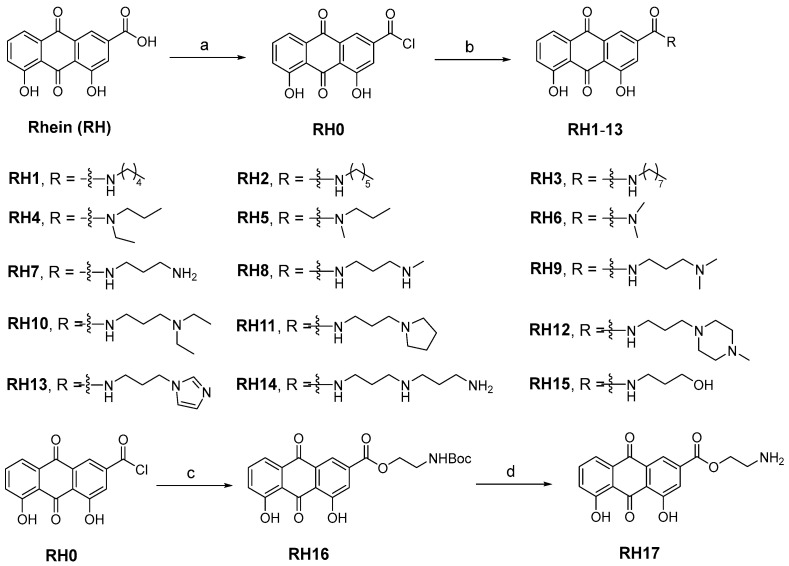
The synthesis of RH derivatives (RH**1**–**17**). Reagents and conditions: (a) DCM (0.01 mM DMF), oxalyl chloride, N_2_, reflux, overnight; (b) anhydrous THF, corresponding amines, r.t., 12–24 h; (c) DCM, TEA, Boc-NH-PEG1-OH, r.t., 3 h; (d) Dioxane, HCl, r.t., 5 h.

The research on the structural modification of RH mainly focuses on the 1,8-dihydroxy and 3-carboxyl positions of RH [14]. According to the analysis of RH, emodin methyl ether, and other homologues, the key component that exerts antimicrobial activity are 1,8-dihydroxy moieties. Therefore, we mainly modify RH by modifying its carboxyl group. The synthetic route of all target compounds, RH**1**–**17**, is described in Figure 1. Rhein was treated with oxalyl chloride at reflux temperature in dichloromethane (DCM) solution to yield intermediate RH**0**. The acetamide derivatives of RH were prepared by adding the corresponding amines into intermediate RH**0**, and the ammonolysis reaction was completed at room temperature. The structures of derivatives (RH**1**–**17**) were characterized by nuclear magnetic resonance and mass spectrometry.

### 2.2. Structure–Activity Relationships of Derivatives

Initially, we assessed the in vitro antibacterial activity of synthetic RH analogs against various *S. aureus* and *E. faecalis* strains by determining their MICs [38]. Gentamicin, a commercially available antimicrobial agent, was used as a reference in this evaluation (Table 1).

To examine the impact of alkyl chains of amines on antibacterial activity, we synthesized the compounds RH**1**, RH**2**, RH**3**, RH**4**, RH**5**, and RH**6**. The clogD_7.4_ values of these compounds were reflective of their lipophilicity, which is also attributed to their relatively weak antibacterial potency. We speculated that the introduced amine groups and the carbonyl group of the backbone would form an acetamide bond, which may improve lipophilicity, inhibit ionization, and exhibit weak antibacterial activity. Based on this, we introduced diamino substituents to explore the influence of ionizable amine on antibacterial activity, and compounds (RH**7**, RH**8**, RH**9**, RH**10**, RH**11**, RH**12**, and RH**13**) were prepared. All compounds showed antibacterial activity equivalent to RH (32 μg/mL), and the MIC values of RH**8** (16 μg/mL) and RH**11** (16 μg/mL) were reduced by one time. By analyzing their physicochemical properties, it was found that the lipophilicity and ionization ability of these compounds were relatively balanced, and finally, their antibacterial effects were improved. The compound RH**14** exhibited a weak antibacterial activity, presumably because its side chain is too long, which leads to too much flexibility to bind to the target. Overall, we used the aminoethanol group to replace the hydroxyl group to obtain compound RH**17**, which has low lipophilicity (1.37) and a high ionization ability (7.65). As a result, compound RH**17** exhibited good antibacterial activity with an MIC of 8 μg/mL. To sum up, we found that lipophilicity and ionization ability are the main properties affecting antibacterial activity.

### 2.3. *RH**17*** Exhibits Antibacterial Activity against Gram-Positive Bacteria

**Figure 2:**
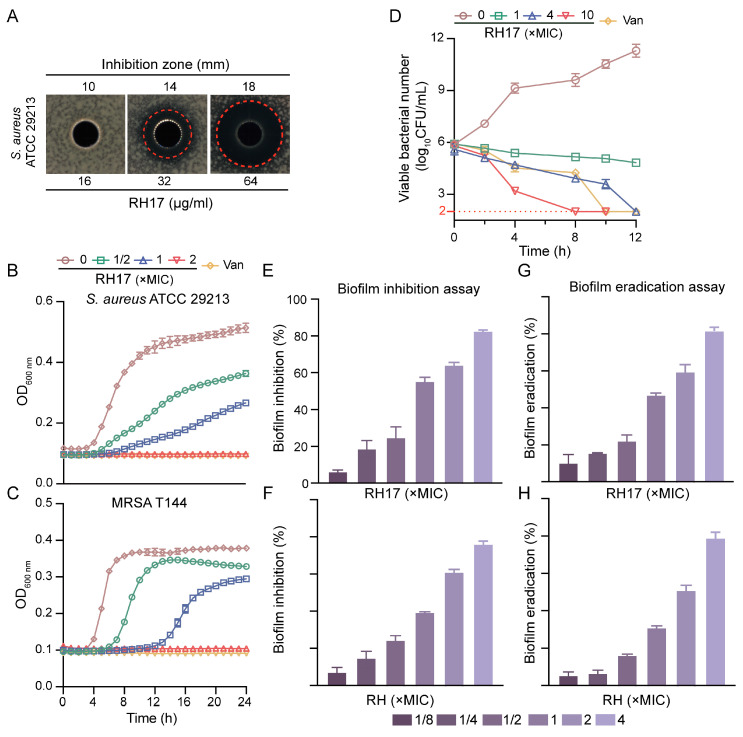
The antibacterial potency of RH**17**. (**A**) Antimicrobial activity of RH**17** against *S. aureus* ATCC 29213. (**B**,**C**) Effect of varying concentrations of RH**17** on growth curves of *S. aureus* isolates. The presented data are depicted as means ± standard deviation (SD). (**B**) *S. aureus* ATCC 29213; (**C**) MRSA T144. (**D**) Time-kill curve of *S. aureus* ATCC 29213 at different concentrations of RH**17**. (**E**–**H**) The antibiofilm activity of RH**17** and RH. (**E**,**F**) RH**17** and RH treatment inhibited biofilm formation on *S. aureus* ATCC 29213 after 24 h. (**E**) RH**17**; (**F**) RH. (**G**,**H**) RH**17** and RH eradicated 24 h pre-formed *S. aureus* ATCC 29213 biofilms. (**G**) RH**17**; (**H**) RH.

In our quest to identify potential antibacterial mechanisms of RH analogs against *S. aureus*, we selected RH**17** as a model compound. RH**17** exhibited inhibitory activity against the standard strain *S. aureus* ATCC 29213, with an MIC of 8 μg/mL, which is four times more potent than RH. Additionally, the antimicrobial activity of RH**17** was assessed using the well diffusion method against *S. aureus* ATCC 29213. The results indicated that RH**17** displayed a significant dose-dependent antibacterial activity against *S. aureus*, with inhibition zones of 10, 14, and 18 mm at concentrations of 8, 16, and 32 μg/mL, respectively (Figure 2A).

To further explore the antibacterial potential, we determined the MICs of both RH and RH**17** against 40 clinical *S. aureus* isolates, which included 20 methicillin-susceptible *S. aureus* (MSSA) and 20 methicillin-resistant *S. aureus* (MRSA). RH**17** exhibited strong antimicrobial activity against MSSA and MRSA isolates, with MIC values ranging from 8 to 16 μg/mL. Specifically, the MIC_50_ and MIC_90_ values for MSSA were both 8 μg/mL, while for MRSA, the MIC_50_ was 8 μg/mL, and MIC_90_ was 16 μg/mL. In comparison, RH showed significantly higher MIC values, ranging from 32 to 128 μg/mL for the same MSSA and MRSA isolates, emphasizing the enhanced potency of RH**17**. Regarding bactericidal activity, the minimum bactericidal concentrations (MBCs) of RH**17** against *S. aureus* isolates ranged from 16 to 64 μg/mL, while RH exhibited higher MBC values, ranging from 64 to >128 μg/mL. These findings indicate that RH**17** exhibits a more muscular bactericidal activity than RH across MSSA and MRSA strains (Table 2 and Table S1). Additionally, RH**17**’s activity was evaluated against *E. faecalis* and vancomycin-resistant *Enterococcus* (VRE). For *E. faecalis*, the MIC_50_ and MIC_90_ values were 16 and 32 μg/mL, respectively. For VRE, the MIC_50_ and MIC_90_ values were both 32 μg/mL (Table 2).

To understand its inhibitory effects on bacterial growth, we conducted growth curve experiments. The data revealed that RH**17** inhibited the growth of planktonic cells of MSSA and MRSA in a dose-dependent manner. While complete inhibition was observed at 2 × MIC, lower concentrations (ranging from 1/2 × MIC to 1 × MIC) showed reduced growth compared to the control but did not achieve full inhibition (Figure 2B,C). These findings underscore RH**17**’s extensive potential against Gram-positive bacteria, encompassing not only *S. aureus* but also *E. faecalis*, another clinically significant pathogen associated with healthcare-associated infections and antibiotic resistance.

### 2.4. Time-Killing Curves of *RH**17*** against S. aureus

The determination of MBCs provided a clearer insight into the bactericidal efficiency of RH**17** against *S. aureus* isolates. To further analyze the time-dependent killing effect of RH**17**, time-kill experiments were conducted on *S. aureus* ATCC 29213, as depicted in Figure 2D. The results visually demonstrate the bactericidal activity of RH**17**. At 1 × MIC, RH**17** exhibited weak bactericidal activity against *S. aureus* ATCC 29213. However, at a concentration of 4 × MIC, no bacterial colonies were observed on the plate after a 24 h incubation at 37 °C. When the RH**17** concentration reached 10 × MIC, the remaining CFU fell to the detection limit within eight hours, indicating that RH**17** kills *S. aureus* in a concentration-dependent manner.

### 2.5. Antibiofilm Activity of *RH**17***

To assess the antibiofilm potential of RH**17**, crystal violet (CV) staining was employed in biofilm formation and eradication assays. *S. aureus* ATCC 29213 was initially examined for its biofilm-forming ability at various concentrations (1/8 × to 4 × MIC) of RH**17** and RH over 24 h. The findings revealed that biofilm formation was inhibited in a concentration-dependent manner (Figure 2E). For RH**17**, significant inhibition was observed at 1/8 × MIC (6.78%), while RH exhibited a slightly lower inhibition rate of 5.34% at the same concentration. At the highest concentration (4 × MIC), RH**17** showed an 82% reduction in biofilm formation, whereas RH demonstrated a comparable inhibition of 78%. Additionally, a biofilm eradication experiment was conducted to evaluate the capacity of RH**17** and RH to disrupt pre-formed 24 h-old biofilms of the same strain. A similar concentration-dependent pattern of biofilm eradication was observed for both compounds (Figure 2F). At 1/8 × MIC, RH**17** eradicated 10.23% of the biofilm, while RH showed an eradication rate of 6.14%. At 4 × MIC, RH**17** demonstrated a biofilm eradication rate of 83%, whereas RH exhibited a slightly lower eradication rate of 81.2%. These results collectively highlight the significant antibiofilm activity of both RH**17** and RH against *S. aureus*, with RH**17** showing marginally better performance in biofilm formation inhibition and eradication at higher concentrations.

### 2.6. Preliminary Antibacterial Mechanism of *RH**17***

**Figure 3:**
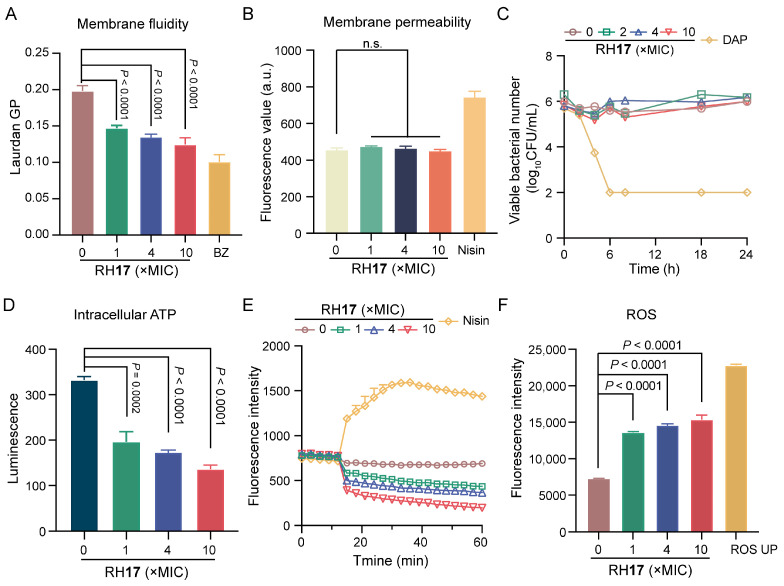
The antibacterial mechanism of RH**17**. (**A**) The fluidity of the membrane was decreased for *S. aureus* ATCC 29213 after treatment with RH**17** for 1 h. Benzyl alcohol (BZ) at 50 mM was used as the positive control. (**B**) There were no effects on the membrane permeability for *S. aureus* ATCC 29213 after treatment with RH**17** for 1 h. Nisin at 200 μg/mL was used as the positive control. (**C**) The effect of RH**17** on the time-kill curves of *S. aureus* ATCC 29213 arrested by cold temperature (0 °C in an ice-water bath) showed no significant impact. Daptomycin (DAP) was used as the positive control, which demonstrated strong bactericidal activity under the same conditions. (**D**) The effect of RH**17** on *S. aureus* ATCC 29213 after 1 h treatment showed a reduction in intracellular ATP levels. (**E**) The effect of RH**17** on *S. aureus* ATCC 29213 showed a decrease in fluorescence intensity. Nisin at 200 μg/mL was used as the positive control. (**F**) The effect of RH**17** on *S. aureus* ATCC 29213 after 1 h treatment resulted in increased ROS production. ROS UP at 500 μg/mL was used as the positive control. A *p*-value of “n.s.” means not significant, suggesting no meaningful difference between groups.

To investigate if the potent bactericidal effect of RH**17** on *S. aureus* is linked to cell membrane damage, a Laurdan fluorescent dye was used to assess its impact on membrane fluidity. Upon treatment with RH**17** at concentrations ranging from 1 × MIC to 10 × MIC, a significant decrease in fluorescence intensity was observed (Figure 3A), indicating reduced membrane fluidity. This reduction can impair the barrier function of bacterial membranes [39]. Subsequently, we assessed cytoplasmic membrane permeability using a propidium iodide (PI) fluorescent dye and detected no significant change in fluorescence intensity (Figure 3B). This suggests that RH**17**’s antibacterial action may operate through mechanisms other than direct bacterial membrane disruption.

In the literature, it has been observed that in a model where bacterial metabolism was inhibited by cold temperature, daptomycin, through its direct action on the bacterial membrane, consistently exhibited bactericidal activity, whereas vancomycin, targeting cell wall synthesis, does not [40]. Subsequently, RH**17** was examined for its effects on the growth of *S. aureus* cultures that were arrested by cold temperature. Time-kill curves demonstrated that daptomycin remained bactericidal against cold-arrested *S. aureus*, whereas RH**17** was not effective (Figure 3C). Those results demonstrated that RH**17** may exert its antibacterial activity by disturbing normal metabolism.

Adenosine triphosphate (ATP), produced by the electronic respiratory chain, is necessary for bacterial metabolism [41]. Therefore, we measured the ATP levels within cells exposed to RH**17** and found that RH**17** significantly reduced intracellular ATP levels (Figure 3D). Previous studies have shown that reduced ATP levels can decrease the proton motive force (PMF) via the F1F0-ATPase [42,43]. We hypothesized that RH**17** might disrupt PMF, which includes the membrane potential (ΔΨ) and the transmembrane proton gradient (ΔpH) [44]. To examine this, we used the fluorescence probe 3,3′-dipropylthiadicarbocyanine iodide (DiSC_3_(5)). When ΔΨ is compromised, DiSC_3_(5) is released from the inner membrane, increasing fluorescence [45,46,47]. Conversely, dissipating ΔpH enhances DiSC_3_(5) uptake, decreasing fluorescence. Our results showed a dose-dependent decrease in fluorescence units, indicating that RH**17** primarily dissipated the ΔpH component of bacterial PMF. Notably, RH**17**, especially at 10 × MIC, significantly disrupted bacterial PMF, potentially leading to reduced ATP synthesis and metabolic disorders. Disrupted membrane homeostasis often leads to the accumulation of reactive oxygen species (ROS). Evidence suggests that ROS formation is crucial in bactericidal antibiotic-mediated killing [48]. Using 2′,7′-dichlorofluorescein diacetate (DCFH-DA) to label bacteria, we found that RH**17** significantly increased ROS production in *S. aureus*. Overall, our results indicate that RH**17** disrupts membrane homeostasis, affects metabolism, and promotes the production of ROS.

## 3. Discussion

MRSA and *Enterococcus* species are increasingly recognized as significant public health threats due to their capacity to develop resistance to antibiotics and cause severe infections [49,50]. Among *Enterococcus* species, *E. faecalis* and *E. faecium* are particularly concerning due to their multidrug-resistant profiles, contributing to severe conditions such as bacteremia and endocarditis [51]. The current rise in resistance among Gram-positive pathogens highlights the need for new antimicrobial agents with novel mechanisms of action [11,52].

In this study, RH**17**, a rhein-based derivative, demonstrated a superior antibacterial activity when compared to the parent compound RH against both *S. aureus* and *Enterococcus* species, including drug-resistant strains like MRSA and VRE. The MIC and MBC values of RH**17** were consistently lower than those of RH, demonstrating RH**17**’s enhanced potency. For instance, RH**17** exhibited MIC values between 8 and 16 μg/mL for *S. aureus* isolates, while the parent RH compound showed significantly higher MIC values, confirming RH**17**’s greater efficacy. The ability of RH**17** to perform well against both *S. aureus* and *Enterococcus* species indicates its broad-spectrum potential against critical Gram-positive pathogens.

Furthermore, our results indicate that RH**17** operates through a multifaceted mechanism of action. It disrupts bacterial membrane stability, causing leakage of intracellular components while simultaneously hindering metabolic processes and increasing the production of ROS. This multifaceted mode of action, targeting both membrane integrity and intracellular metabolic processes, accelerates bacterial death and may also reduce the likelihood of resistance development, as it differs from traditional antibiotics that typically focus on a single target pathway [53].

While these in vitro findings are promising, several limitations exist. First, although RH**17** has shown potent activity against both *S. aureus* and *Enterococcus* species, the exact molecular targets within these pathogens remain unidentified. Future studies should focus on elucidating these targets through proteomic and metabolomic analyses. Additionally, the current study did not assess RH**17**’s efficacy in vivo, making it necessary to conduct animal model studies to evaluate its pharmacokinetics, bioavailability, and safety profile. These preclinical evaluations are essential for determining the therapeutic potential of RH**17**. Furthermore, the potential applications of RH**17** may extend beyond *S. aureus* and Enterococcus species. Given its novel mechanism of action and strong efficacy against Gram-positive pathogens, RH**17** could be investigated for activity against other multidrug-resistant organisms. Expanding the spectrum of testing to include various resistant Gram-positive bacteria will help establish RH**17**’s role as a broad-spectrum antimicrobial agent.

In conclusion, we have developed RH**17**, a potent rhein derivative with a significant antimicrobial activity against both *S. aureus* and *Enterococcus* species. The dual mechanism of action of RH**17**, which disrupts both bacterial membrane integrity and metabolism, makes it a promising candidate for further development. However, additional studies, including in vivo testing and target identification, are necessary to explore its therapeutic potential fully and address our study’s current limitations.

## 4. Materials and Methods

### 4.1. Materials

Rhein and other chemical reagents and solvents were sourced from Shanghai Titan Scientific Co., Ltd., Shanghai, China. Nuclear magnetic resonance (NMR) spectra were obtained in deuterated dimethyl sulfoxide (DMSO_-_*d*_6_) and deuterated methanol (CD_3_OD) using a Bruker Avance 400 MHz or 600 MHz instrument (Billerica, MA, USA) with TMS as the internal standard. High-resolution mass spectra (HRMS) were recorded on an Agilent LC-MS/MS (Santa Clara, CA, USA). Thin-layer chromatography (TLC) analyses were conducted on precoated silica-gel F254 plates with solvent systems of petroleum/ethyl acetate (6:4) and dichloromethane/methanol (9:1). Silica-gel (100–200 mesh) was used in column chromatography for purification of derivatives. Additionally, a preparative Shimadzu HPLC equipped with a C18 column (Shim-pack GIST, C18, 5 μm, 10 × 250 mm, Kyoto, Japan) was used to purify the derivatives, employing a methanol/distilled water (10–100%) mobile phase containing 0.1% formic acid.

### 4.2. Synthesis of RH Derivatives

*N*-pentyl-4,5-dihydroxyl-9,10-dioxo-9,10-dihydroanthracene-2-carboxamide (RH**1**): a solution of RH (400 mg, 1.41 mmol) and dimethylformamide (DMF, 0.1 mM) in 6 mL dichloromethane (DCM) was added to 1.8 mL oxalyl chloride (2.0 M in dichloromethane, 2 mL) under nitrogen at 0 °C. The reaction mixture was then brought to room temperature and refluxed at 42 °C for eight hours. The resulting crude product (RH**0**) was unstable and directly used in the next reaction without further purification. The crude product (100 mg, 0.33 mmol) was dissolved in anhydrous THF (3 mL) along with amylamine (3 mL) and stirred for 12 h at room temperature. The reaction mixture was then cooled to room temperature and concentrated under reduced pressure to remove the solvent. The crude product was purified by silica gel column chromatography and eluted with a gradient of DCM/methanol (100:1, *v*/*v*) to obtain RH**1** (42 mg). ^1^H NMR (600 MHz, DMSO-*d*_6_) *δ*: 8.88 (t, 1H, *J* = 5.5 Hz, -NH-), 8.12 (d, 1H, *J* = 1.7 Hz, Ar-H), 7.83 (m, 1H, Ar-H), 7.76 (m, 2H, 2×Ar-H), 7.41 (dd, 1H, *J* = 8.3, 1.2 Hz, Ar-H), 3.27 (m, 2H, -CH_2_-), 1.55 (m, 2H, -CH_2_-), 1.31 (m, 4H, 2×-CH_2_-), and 0.87 (m, 3H, CH_3_). HR-ESI-MS *m/z*: calculated for C_20_H_20_NO_5_ [M+H]^+^ 354.1336; 354.1336 was observed.

*N*-hexyl-4,5-dihydroxyl-9,10-dioxo-9,10-dihydroanthracene-2-carboxamide (RH**2**): the title compound was prepared using a procedure analogous to that used for compound RH**1**, starting from compound RH**0** and hexylamine. The resultant compound RH**2** was isolated as a yellow powder, with a yield of 67%. ^1^H NMR (600 MHz, DMSO-*d*_6_) *δ*: 8.89 (t, 1H, *J* = 5.5 Hz, -NH-), 8.13(d, 1H, *J* = 1.5 Hz, Ar-H), 7.84 (m, 1H, Ar-H), 7.76 (m, 2H, 2×Ar-H), 7.41 (dd, 1H, *J* = 8.3, 1.1 Hz, Ar-H), 3.28 (m, 2H, -CH_2_-), 1.98 (m, 2H, -CH_2_-), 1.54 (m, 2H, -CH_2_-), 1.29 (m, 4H, 2×-CH_2_-), and 0.86 (m, 3H, CH_3_). HR-ESI-MS *m/z*: calculated for C_21_H_22_NO_5_ [M+H]^+^ 368.1492; 368.1492 was observed.

*N*-heptyl-4,5-dihydroxyl-9,10-dioxo-9,10-dihydroanthracene-2-carboxamide (RH**3**): the title compound was prepared using a method similar to that for compound RH**1**, starting from compound RH**0** and octylamine. Compound RH**3** was isolated as a yellow powder with a yield of 56%. ^1^H NMR (600 MHz, DMSO-*d*_6_) *δ*: 8.87 (t, 1H, *J* = 5.4 Hz, -NH-), 8.12 (s, 1H, Ar-H), 7.83 (t, 1H, *J* = 7.9 Hz, Ar-H), 7.75 (m, 2H, 2×Ar-H), 7.41 (d, 1H, *J* = 8.4 Hz, Ar-H), 3.28 (m, 2H, -CH_2_-), 1.99(m, 2H, -CH_2_-), 1.54 (m, 2H, -CH_2_-), 1.28(m, 8H, 4×-CH_2_-), and 0.85 (m, 3H, CH_3_). HR-ESI-MS *m/z*: calculated for C_23_H_26_NO_5_ [M+H]^+^ 396.1805; 396.1802 was observed.

*N*-ethyl-4,5-dihydroxy-9,10-dioxo-*N*-propyl-9,10-dihydroanthracene-2-carboxamide (RH**4**): the title compound was prepared using the same method as that for RH**1**, starting with compound RH**0** and *N*-ethylpropan-1-amine. Compound RH**4** was obtained as a yellow powder with a yield of 62%. ^1^H NMR (400 MHz, CD_3_OD) *δ*: 7.91 (s, 1H, Ar-H), 7.64 (s, 1H, Ar-H), 7.55 (m, 2H, 2×Ar-H), 7.20 (dd, 1H, *J* = 5.4, 2.1 Hz, Ar-H), 3.31 (m, 2H, -CH_2_-), 3.18 (m, 2H, -CH_2_-), 1.64 (m, 2H, -CH_2_-), 1.16 (t, 3H, *J* = 8.0 Hz, CH_3_), and 0.87 (t, 3H, *J* = 7.5 Hz, CH_3_). ^13^C NMR (100 MHz, CD_3_OD) *δ*: 193.3, 181.8, 167.4, 163.6, 163.3, 142.6, 138.8, 134.9, 134.4, 125.8, 123.8, 120.9, 118.7, 118.5, 116.8, 50.8, 48.3, 38.2, 25.2, and 9.1. HR-ESI-MS *m/z*: calculated for C_20_H_20_NO_5_ [M+H]^+^ 354.1336; 354.1328 was observed.

*N*-propyl-4,5-dihydroxy-9,10-dioxo-*N*-propyl-9,10-dihydroanthracene-2-carbox-amide (RH**5**): the title compound was prepared using the same method as that for RH**1**, starting from compound RH**0** and *N*-methylpropylamine. Compound RH**5** was obtained as a yellow powder with a yield of 58%. ^1^H NMR (400 MHz, CD_3_OD) *δ*: 7.74 (m, 3H, 3×Ar-H), 7.31 (m, 1H, 2×Ar-H), 3.56, 3.27 (m, 0.8H and 1.2 H, -CH_2_-), 3.10, 3.00 (s, 1.5H and 1.5H, CH_3_), 1.75 and 1.65 (m, 1H and 1H, -CH_2_-), and 1.02 and 0.81 (m, 1.5H and 1.5H, CH_3_). ^13^C NMR (100 MHz, CD_3_OD) *δ*: 193.8, 182.3, 170.8, 163.7, 163.6, 146.2, 138.7, 135.6, 134.9, 125.8, 122.9, 120.9, 118.3, 117.7, 117.1, 37.7, 33.1, 22.4, and 11.5. HR-ESI-MS *m/z*: calculated for C_19_H_18_NO_5_ [M+H]^+^ 340.1179; 340.1165 was observed.

*N*,*N*-dimethyl-4,5-dihydroxy-9,10-dioxo-9,10-dihydroanthracene-2-carboxamide (RH**6**): the title compound was prepared following the same method as that for RH**1**, starting from compound RH**0** and dimethylamine. Compound RH**6** was obtained as a yellow powder with a yield of 64%. ^1^H NMR (400 MHz, DMSO-*d*_6_) *δ*: 11.94 (brs, 2H, 2×-OH), 7.83 (t, 1H, *J* = 7.8 Hz, Ar-H), 7.73 (d, 1H, *J* = 7.2 Hz, Ar-H), 7.63 (d, 1H, *J* = 1.3 Hz, Ar-H), 7.41 (d, 1H, *J* = 7.4 Hz, Ar-H), 7.38 (d, 1H, *J* = 1.2 Hz, Ar-H), 3.02 (s, 3H, CH_3_), and 2.92 (s, 3H, CH_3_). ^13^C NMR (100 MHz, DMSO-*d*_6_) *δ*: 191.37, 181.0, 167.5, 161.3, 161.1, 144.7, 137.4, 133.7, 133.2, 124.5, 121.7, 119.3, 117.0, 116.3, 116.0, 38.6, and 34.6. HR-ESI-MS *m/z*: calculated for C_17_H_14_NO_5_ [M+H]^+^ 312.0866; 312.0864 was observed.

*N*-(3-aminopropyl)-4,5-dihydroxy-9,10-dioxo-9,10-dihydroanthracene-2-carbox-amide (RH**7**): the title compound was prepared using the same synthetic procedure as that for compound RH**1**, starting from compound RH**0** and 1,3-diaminopropane. Compound RH**7** was obtained as a yellow powder with a yield of 36%. ^1^H NMR (400 MHz, CD_3_OD) *δ*: 8.20 (d, 1H, *J* = 1.6 Hz, Ar-H), 7.84 (dd, 1H, *J* = 7.7, 1.5 Hz, Ar-H), 7.79 (m, 1H, Ar-H), 7.75 (d, 1H, *J* = 1.6 Hz, Ar-H), 7.37 (dd, 1H, *J* = 8.1, 1.5 Hz, Ar-H), 3.53 (t, 2H, *J* = 6.8 Hz, -CH_2_-), 3.02 (t, 2H, *J* = 7.0 Hz, -CH_2_-), and 1.99 (m, 2H, -CH_2_-). HR-ESI-MS *m/z*: calculated for C_18_H_17_N_2_O_5_ [M+H]^+^ 341.1132; 341.1135 was observed.

*N*-(3-(methylamino)propyl)-4,5-dihydroxy-9,10-dioxo-9,10-dihydroanthracene-2-carboxamide (RH**8**): the title compound was prepared following the same synthetic procedure as that for RH**1**, starting from compound RH**0** and *N*-methyl-1,3-propanediamine. The final product, compound RH**8**, was obtained as a yellow powder with a yield of 31%. ^1^H NMR (400 MHz, CD_3_OD) *δ*: 8.15 (s, 1H, Ar-H), 7.77 (m, 2H, 2×Ar-H), 7.71 (s, 1H, Ar-H), 7.34 (dd, 1H, *J* = 7.2, 2.1 Hz, Ar-H), 3.53 (t, 2H, *J* = 6.8 Hz, -CH_2_-), 3.08 (t, 2H, *J* = 7.4 Hz, -CH_2_-), 2.74 (s, 3H, -CH_3_), and 2.02 (m, 2H, -CH_2_-). ^13^C NMR (100 MHz, CD_3_OD) *δ*: 193.9, 182.4, 168.2, 163.8, 163.6, 142.8, 138.7, 135.4, 134.9, 125.8, 123.9, 120.9, 119.0, 118.7, 117.3, 48.0, 37.8, 33.7, and 27.6. HR-ESI-MS *m/z*: calculated for C_19_H_19_N_2_O_5_ [M+H]^+^ 355.1288; 355.1280 was observed.

*N*-(3-(dimethylamino)propyl)-4,5-dihydroxy-9,10-dioxo-9,10-dihydroanthracene-2-carboxamide (RH**9**): the title compound was prepared using the same synthetic procedure as that for RH**1**, starting from compound RH**0** and *N*,*N*-dimethyl-1,3-propanediamine. The final product, RH**9**, was obtained as a yellow powder with a yield of 33%. ^1^H NMR (400 MHz, DMSO-*d*_6_) *δ*: 9.03 (t, 1H, -NH-), 8.13 (d, 1H, *J* = 1.6 Hz, Ar-H), 7.84 (t, 1H, *J* = 7.6 Hz, Ar-H), 7.77 (d, 1H, *J* = 1.6 Hz, Ar-H), 7.76 (dd, 1H, *J* = 7.6, 1.0 Hz, Ar-H), 7.42 (dd, 1H, *J* = 7.6, 1.0 Hz, Ar-H), 3.07 (m, 2H, -CH_2_-), 2.76 (s, 6H, 2×CH_3_), and 1.90 (m, 2H, -CH_2_-). ^13^C NMR (150 MHz, DMSO-*d*_6_) *δ*: 191.5, 181.3, 164.4, 161.5, 161.2, 141.5, 137.7, 133.7, 133.4, 124.7, 122.5, 119.5, 117.8, 117.5, 116.2, 54.9, 46.4, 2 × 42.5, 36.7, and 24.4. HR-ESI-MS *m/z*: calculated for C_20_H_21_N_2_O_5_ [M+H]^+^ 369.1445; 369.1441 was observed.

*N*-(3-(diethylamino)propyl)-4,5-dihydroxy-9,10-dioxo-9,10-dihydroanthracene-2-carboxamide (RH**10**): the title compound was synthesized following the same procedure as that for RH**1**, using RH**0** and 3-diethylaminopropylamine. The resulting product, RH**10**, was acquored as a yellow powder with a yield of 42%. ^1^H NMR (400 MHz, CD_3_OD) *δ*: 7.94 (s, 1H, Ar-H), 7.64 (m, 1H, Ar-H), 7.58 (m, 2H, 2×Ar-H), 7.20 (d, 1H, *J* = 7.0 Hz, Ar-H), 3.53 (m, 2H, -CH_2_-), 3.31 (m, 6H, 2×-CH_2_-), 2.11 (m, 2H, -CH_2_-), and 1.37 (t, 6H, *J* = 7.3 Hz, 2×CH_3_). ^13^C NMR (100 MHz, CD_3_OD) *δ*: 193.3, 181.8, 167.4, 163.6, 163.3, 142.5, 138.7, 135.0, 134.4, 130.8, 125.8, 123.8, 120.9, 118.7, 118.5, 116.8, 50.9, 38.2, 30.7, 2 × 25.3, and 2 × 9.2. HR-ESI-MS *m/z*: calculated for C_22_H_25_N_2_O_5_ [M+H]^+^ 397.1758; 397.1750 was observed.

*N*-(3-(diethylamino)propyl)-4,5-dihydroxy-9,10-dioxo-9,10-dihydroanthracene-2-carboxamide (RH**11**): the title compound was synthesized following the same procedure as that for RH**1**, starting from RH**0** and 1-(3-aminopropyl) pyrrolidine. The resulting product, RH**11**, was obtained as a yellow powder with a yield of 37%. ^1^H NMR (600 MHz, DMSO-*d*_6_) *δ*: 8.98 (m, 1H, -NH-), 8.28 (s, 1H, Ar-H), 8.03 (s, 1H, Ar-H), 7.77 (m, 1H, Ar-H), 7.68 (m, 2H, 2×Ar-H), 3.34 (m, 2H, -CH_2_-), 2.82 (m, 4H, 2×-CH_2_-), 2.79 (m, 2H, -CH_2_-), 1.83 (m, 2H, -CH_2_-), and 1.80 (m, 4H, 2×-CH_2_-). HR-ESI-MS *m/z*: calculated for C_22_H_23_N_2_O_5_ [M+H]^+^ 395.1601 and observed 395.1611.

*N*-(3-(4-methylpiperazin-1-yl)propyl)-4,5-dihydroxy-9,10-dioxo-9,10-dihydro-anthracene-2-carboxamide (RH**12**): the title compound was synthesized following the same procedure as that for RH**1**, starting from RH**0** and 1-(3-aminopropyl)-4-methylpiperazine. The resulting product, RH**12**, was obtained as a yellow powder with a yield of 41%. ^1^H NMR (400 MHz, CD_3_OD) *δ*: 8.50 (s, 2H, -NH- and -OH), 8.15 (d, 1H, *J* = 1.7 Hz, Ar-H), 7.82 (m, 1H, Ar-H), 7.78 (m, 1H, Ar-H), 7.72 (d, 1H, *J* = 1.7 Hz, Ar-H), 7.36 (dd, 1H, *J* = 8.0, 1.6 Hz, Ar-H), 3.49 (m, 2H, -CH_2_-), 2.92 (m, 4H, 2× -CH_2_-), 2.74 (m, 4H, 2× -CH_2_-), 2.64 (m, 2H, -CH_2_-), 2.60 (s, 3H, CH_3_), and 1.88 (m, 2H, -CH_2_-). HR-ESI-MS *m/z*: calculated for C_23_H_26_N_3_O_5_ [M+H]^+^ 424.1867; 424.1863 was observed.

*N*-(3-(1H-imidazol-1-yl)propyl)-4,5-dihydroxy-9,10-dioxo-9,10-dihydro-anthra-cene-2-carboxamide (RH**13**): the title compound was synthesized by applying the same method used for RH**1**, starting from RH**0** and 1-(3-aminopropyl)-4-methylpiperazine. The product, RH**13**, was obtained as a yellow powder with a yield of 36%. ^1^H NMR (400 MHz, CD_3_OD) *δ*: 8.18 (d, 1H, *J* = 1.6 Hz, Ar-H), 7.92 (s, 1H, Ar-H), 7.83 (m, 1H, Ar-H), 7.79 (m, 1H, Ar-H), 7.72 (d, 1H, *J* = 1.6 Hz, Ar-H), 7.36 (dd, 1H, *J* = 8.1, 1.3 Hz, Ar-H), 7.29 (s, 1H, Ar-H), 7.08 (s, 1H, Ar-H), 4.18 (t, 2H, *J* = 7.0 Hz, -CH_2_-), 3.44 (t, 2H, *J* = 6.7 Hz, -CH_2_-), and 2.02 (m, 2H, -CH_2_-). HR-ESI-MS *m/z*: calculated for C_21_H_18_N_3_O_5_ [M+H]^+^ 392.1241; 392.1238 was observed.

*N*-(3-((3-aminopropyl)amino)propyl)-4,5-dihydroxy-9,10-dioxo-9,10-dihydro-anthracene-2-carboxamide (RH**14**): the title compound was synthesized following the same procedure as that for compound RH**1**, using RH**0** and bis(3-aminopropyl)amine. The resulting compound, RH**14**, was obtained as a yellow powder with a yield of 31%. ^1^H NMR (400 MHz, CD_3_OD) *δ*: 8.15 (d, 1H, *J* = 1.4 Hz, Ar-H), 7.78 (m, 2H, 2×Ar-H), 7.72 (d, 1H, *J* = 1.4 Hz, Ar-H), 7.34 (dd, 1H, *J* = 7.6, 1.4 Hz, Ar-H), 3.54 (t, 2H, *J* = 6.8 Hz, -CH_2_-), 3.06 (q, 4H, *J* = 7.5 Hz, 2×-CH_2_-), 3.01 (t, 2H, *J* = 7.5 Hz, -CH_2_-), and 2.01 (m, 4H, 2×-CH_2_-). HR-ESI-MS *m/z*: calculated for C_21_H_24_N_3_O_5_ [M+H]^+^ 398.1710 and observed 398.1710.

*N*-(3-hydroxypropyl)-4,5-dihydroxy-9,10-dioxo-9,10-dihydro-anthracene-2-carboxamide (RH**15**): the title compound was synthesized using the same procedure as that for compound RH**1**, starting from compound RH**0** and 3-aminopropan-1-ol. Compound RH**15** was obtained as a yellow powder with a yield of 52%. ^1^H NMR (600 MHz, DMSO-*d*_6_) *δ*: 8.87 (t, 1H, *J* = 5.6 Hz, -NH-), 8.12 (d, 1H, *J* = 1.5 Hz, Ar-H), 7.83 (t, 1H, *J* = 5.2 Hz, Ar-H), 7.75 (m, 2H, 2×Ar-H), 7.41 (d, 1H, *J* = 5.2 Hz, Ar-H), 3.48 (t, 2H, *J* = 4.1 Hz, -CH_2_-), 3.35 (t, 2H, *J* = 6.7 Hz, -CH_2_-), and 1.71 (m, 2H, -CH_2_-). ^13^C NMR (150 MHz, DMSO-*d*_6_) *δ*: 191.4, 181.2, 164.0, 161.5, 161.3, 141.8, 137.5, 133.6, 133.4, 129.6, 124.6, 122.5, 119.4, 117.5, 116.2, 58.5, 36.9, and 32.2. HR-ESI-MS *m/z*: calculated for C_18_H_16_NO_6_ [M+H]^+^ 342.0972; 342.0968 was observed.

2-Aminoethyl-4,5-dihydroxy-9,10-dioxo-9,10-dihydroanthracene-2-carboxylate (RH**17**): *N*-Boc-ethanolamine (81 mg, 0.5 mmol) was introduced to a solution containing RH**0** (100 mg, 0.33 mmol) in DCM. The reaction mixture was stirred at room temperature, and triethylamine (TEA, 51 mg, 0.5 mmol) was gradually added. After three hours, the solvent was evaporated under reduced pressure, and the product was purified using column chromatography on a silica gel with a petroleum ether (PE)/ethyl ether (EA) (4:1, *v*/*v*) solvent system, resulting in RH**16** (115 mg) with an 82% yield. To remove the Boc-protected group, 4 M HCl in dioxane (1.0 mL) was added dropwise to a solution of RH**16** (43 mg, 0.10 mmol) in dioxane (2.0 mL) with vigorous stirring at room temperature. After five hours, the solvent was evaporated under reduced pressure, and the crude product was washed with EA, yielding RH**17** (30 mg) with a 92% yield. ^1^H NMR (400 MHz, DMSO-*d*_6_) *δ*: 11.97 (br s, 1H, -OH), 11.86 (s, 1H, -OH), 8.29 (br s, 2H, -NH), 8.20 (m, 1H, Ar-H), 8.05 (m, 1H, Ar-H), 7.84 (s, 1H, Ar-H), 7.74 (m, 1H, Ar-H), 7.43 (m, 1H, Ar-H), 4.54 (t, 2H, *J* = 4.9 Hz, -CH_2_-), and 3.27 (m, 2H, -CH_2_-). ^13^C NMR (150 MHz, DMSO-*d*_6_) *δ*: 191.3, 180.9, 163.9, 161.4, 161.0, 137.7, 136.2, 133.8, 133.2, 124.8, 124.7, 119.5, 119.1, 118.9, 116.2, 62.4, and 37.8. HR-ESI-MS *m/z*: calculated for C_17_H_14_NO_6_ [M+H]^+^ 328.0816; 328.0813 was observed.

### 4.3. Antibacterial Susceptibility Assay

Broth microdilution method: derivatives were dissolved in DMSO, followed by a two-fold serial dilution in Mueller–Hinton Broth (MHB) to achieve final concentrations ranging from 128 to 0.25 μg/mL. Each well in the microtiter plate contained 100 μL of the diluted compounds and 100 μL of a bacterial suspension at a final concentration of 10^6^ colony-forming units (CFU)/mL, prepared from an overnight culture in Brain Heart Infusion Broth (BHI). Plates were incubated at 37 °C for 16 h. The negative control well contained 100 μL of MHB alone, while the positive well contained 100 μL of MHB with the bacterial suspension. The MIC values were identified as the lowest concentrations without visible bacterial growth. MICs were verified through three independent biological experiments.

Well diffusion method: a cell suspension of the tested pathogenic cultures (final concentration 10^6^ CFU/mL) was spread over the surface of a Mueller–Hinton (MH) agar plate. Each well (with an inner diameter of 6 mm) was filled with 100 μL of the RH**17** at 1×, 2×, 4×, and 8 × MIC. Plates were incubated at 37 °C for 16 h. Antimicrobial activity was assessed by measuring the diameter of the clear inhibition zone around each well.

### 4.4. Minimum Bactericidal Concentration

Minimum bactericidal concentrations (MBC) are the minimum concentration of a compound required to kill an organism. Tested strains were cultured, and cell density was adjusted before exposing the cultures to varying concentrations of RH**17** for 24 h. Post incubation, the samples were plated on MH agar and further incubated at 37 °C for 24 h to monitor growth. The MBC of RH**17** was determined as the lowest concentration at which no organism growth was observed. Experiments were conducted in triplicates.

### 4.5. Bactericidal Kinetics Assay

The procedure was adapted from previously reported methods. Briefly, bacterial suspensions were prepared and diluted to a final concentration of 10^6^ CFUs/mL in MHB. RH**17** solutions were then added to the bacterial suspensions to achieve final concentrations of 0 × MIC (as the negative control, using PBS), 1 × MIC, 4 × MIC, and 10 × MIC. Vancomycin at 10 × MIC was used as the positive control. The cultures were incubated in a shaking incubator at 37 °C. At time points 0, 0.25, 0.5, 1, 2, 4, 8, 12, and 24 h, 100 μL aliquots of the culture were diluted appropriately and spread on agar plates. Following an 18 h incubation at 37 °C, colonies on the plates were counted and quantified as CFU/mL.

To assess the time-kill kinetics under metabolic inhibition, the bacterial suspension was pre-incubated at 0 °C in an ice-water bath to inhibit metabolism. Afterward, the prepared bacterial suspension was treated with RH**17** at concentrations of 0 × MIC (as the negative control, using PBS), 1 × MIC, 4 × MIC, and 10 × MIC. The cultures were maintained at 0 °C in the ice-water bath throughout the experiment. Sampling and subsequent colony counting were conducted as described previously for the standard time-kill assay.

### 4.6. Anti-Biofilm Assay

The anti-biofilm assay was performed following the literature with minor modifications [54,55]. A biofilm inhibition assay was performed in a 96-well microplate. Bacterial isolates were cultured overnight in tryptic soy broth (TSB) containing 0.25% glucose at 37 °C. The bacterial suspension was then adjusted to match the turbidity of a 0.5 McFarland standard. Bacterial aliquots of 200 μL with different concentrations of RH**17** were added to each microplate well and incubated at 37 °C for 24 h. Negative control wells contained only broth. After incubation, the wells were centrifugated, gently washed with PBS, fixed with 99% methanol, and allowed to dry at room temperature. The attached biofilm cells were stained using 0.1% crystal violet, and the dye was subsequently dissolved with 33% glacial acetic acid. Optical density (OD) at 570 nm was measured using a microplate reader. The biofilm eradication assay is similar to the biofilm inhibition assay. First, a mature biofilm was formed using the above method; the spent culture medium was removed, and the biofilm was washed with PBS three times. Then the biofilms were exposed to different concentrations of RH**17** for 24 h. The control group was added with the same amount of PBS solution. Finally, staining and measuring were carried out according to the above protocols.

### 4.7. Membrane Fluidity Assay

Membrane fluidity was evaluated using Laurdan generalized polarization (GP) (MCE, Monmouth Junction, NJ, USA, Cat No. HY-D0080) based on previously published protocols with modifications [56]. Overnight cultures of *S. aureus* were diluted to an OD_600_ of 0.5 and treated with RH**17** at 1×, 4×, and 10 × MIC. After one hour of incubation, the cells were washed twice with PBS, resuspended to the original volume, and stained with 10 μM Laurdan for 20 min at room temperature. Subsequently, the cells were washed three times with PBS and transferred to clear, flat-bottomed 96-well black plates. Laurdan fluorescence was recorded at 460 and 500 nm with excitation at 330 nm. Laurdan GP values were calculated using the formula: GP = (I_460_ − I_500_)/ (I_460_ + I_500_). PBS was used as the negative control, while 50 mM benzyl alcohol (BZ) was employed as the positive control.

### 4.8. Membrane Permeability Assay

Propidium iodide (PI, Sigma-Aldrich, St. Louis, MO, USA, Cat No. 537079) staining was used to assess bacterial membrane permeability as described in previous studies [57]. Briefly, bacterial cultures were diluted to OD_600_ = 0.5 and exposed to RH**17** at 1 ×, 4 ×, and 10 × MIC concentrations. Negative controls (PBS) and positive controls (200 μg/mL nisin) were included in parallel. After one hour of treatment, 10 μM PI stain was added and incubated for 15 min. The cultures were washed twice with PBS, and fluorescence (Ex/Em = 540/610 nm) was measured within one hour using a microplate reader (TECAN Infinite 200 pro, Mennedorf, Switzerland).

### 4.9. ATP Determination Assay

The ATP levels of *S. aureus* ATCC 29213 were assessed using an Enhanced ATP Assay Kit (Beyotime, Shanghai, China, Cat No. S0027) as described previously [58]. In brief, overnight bacterial cultures were sub-cultured for 2–3 h at 37 °C. The cultures were then washed and resuspended in PBS to reach an OD_600_ of 0.5. Following this, the bacterial suspensions were exposed to different concentrations of RH**17** for one hour. After treatment, the suspensions were centrifuged, and the supernatants were collected to measure extracellular ATP levels. Concurrently, the bacterial pellets were lysed, centrifuged, and the resulting supernatants were used to determine intracellular ATP levels. Chemiluminescence was measured using the TECAN Infinite 200 pro microplate reader.

### 4.10. Membrane Depolarization Assay

Membrane depolarization was assessed using 3,3′-dipropylthiadicarbocyanine iodide [DiSC_3_(5)] (MCE, Cat No. HY-D0085), following previously described methods with modifications [59]. Briefly, overnight bacterial cultures were diluted to an OD_600_ of 0.5 and treated with RH**17** at concentrations of 1×, 4×, and 10 × MIC. HEPES buffer (5 mM, 20 mM glucose, pH 7.4) was used as the negative control, and 200 μg/mL nisin served as the positive control. After one hour of incubation, DiSC_3_(5) was added to a final concentration of 10 μM and incubated for 30 min. Fluorescence (Ex/Em = 622/670 nm) was measured using a microplate reader (TECAN Infinite 200 pro) to assess membrane depolarization.

### 4.11. Reactive Oxygen Species (ROS) Determination Assay

The ROS Assay Kit (Beyotime, Cat No. S0033S) measured the ROS levels. Bacterial cells were diluted to an OD_600_ of 0.5, treated with RH**17** at 1×, 4×, and 10 × MIC, and incubated for one hour. After centrifugation, the supernatant was collected and DCFDA (100 μM) was added. Following a 30 min incubation, fluorescence was measured at Ex/Em = 485/535 nm. ROS UP was the positive control, and PBS was the negative control.

### 4.12. Statistical Analysis

Statistical analyses were conducted using Prism version 9.0. Data were expressed as mean ± standard deviation (SD). Comparisons between groups were performed using two-way ANOVA, with the level of significance set at a *p*-value of 0.05 or less.

## Figures and Tables

**Table 1:** In vitro antimicrobial activities against *S. aureus* and *E. faecalis* and the physicochemical properties of RH derivatives (RH**1**–**17**).

Com.	clogD_7.4_^1^	pKa ^2^	Gram-Positive (μg/mL)	
			*S. aureus* ATCC 29213	*E. faecalis* ATCC 29212
RH	1.89	4.31	32	64
RH**1**	2.85	6.15	32	64
RH**2**	3.09	6.15	>128	>128
RH**3**	3.49	6.15	64	>128
RH**4**	2.73	6.15	64	>128
RH**5**	2.56	6.15	64	>128
RH**6**	2.06	6.14	32	64
RH**7**	0.77	7.37	32	64
RH**8**	1.41	7.37	16	32
RH**9**	2.02	7.37	32	32
RH**10**	2.40	7.37	32	32
RH**11**	2.33	7.37	16	32
RH**12**	2.73	7.37	32	64
RH**13**	1.92	6.80	64	>128
RH**14**	0.10	8.81	128	>128
RH**15**	1.92	6.15	64	>128
RH**17**	1.37	7.65	8	16
GEN ^3^	/	/	0.25	2

^1^ clogD_7.4_ refers to the calculated logD at pH = 7.4, which represents the lipophilicity of compounds. ^2^ pKa represents the acid dissociation constant, which represents the ionization ability of compounds. ^3^ GEN refers to gentamicin.

**Table 2:** Antimicrobial activity of RH**17** against *S. aureus* and *E. faecalis* isolates.

Strains	No.	MIC Distribution (μg/mL)			MIC_50_^1^/MIC_90_^2^ (μg/mL)
		8	16	32	
MSSA	20	18	2	/	8/8
MRSA	20	12	8	/	8/16
*E. faecalis*	20	/	13	7	16/32
VRE ^3^	20	/	8	12	32/32

^1^ MIC_50_ refers to the concentration that would inhibit the growth of 50% of the tested bacterial isolates. ^2^ MIC_90_ refers to the concentration that would inhibit the growth of 90% of the tested bacterial isolates. ^3^ VRE, vancomycin-resistant *Enterococcus*.

## Data Availability

Data are contained within the article and Supplementary Materials.

## Supplementary Materials

The following supporting information can be downloaded at https://www.mdpi.com/article/10.3390/antibiotics13090882/s1, Figures S1–S30: the NMR spectrum data and HR-ESI-MS spectrum data of compounds RH**1**–**17**; Figure S31: Cytotoxicity of RH and RH**17**; Table S1: MIC and MBC values of RH and RH**17** against *S. aureus* isolates (n = 40).
