# Supplementary material for: A Rhein-Based Derivative Targets Staphylococcus aureus
Source: Antibiotics (Basel). 2024 Sep 13;13(9):882. doi: 10.3390/antibiotics13090882 (PMC11428220; doi:10.3390/antibiotics13090882)
Supplement: Supplementary file 1 [file antibiotics-13-00882-s001.zip › antibiotics-3167904-supplementary.pdf]

A Rhein-Based Derivative Targets *Staphylococcus aureus*

Xiaojia Liu, Yuan Liu, Meirong Song, Kui Zhu*, Jianzhong Shen*

National Key Laboratory of Veterinary Public Health and Safety, College of Veterinary Medicine, China Agricultural University, No.2 Yuanmingyuan West Road, Beijing 100193, China; liuxj@cau.edu.cn (X.L.); sy20233051144@cau.edu.cn (Y.L.); meirong_song@cau.edu.cn (M.S.)

*Corresponding author: Prof. Kui Zhu; Prof. Jianzhong Shen.
Email: zhuk@cau.edu.cn; sjz@cau.edu.cn.

This PDF file includes:

Figures S1 to S31.

Table S1.

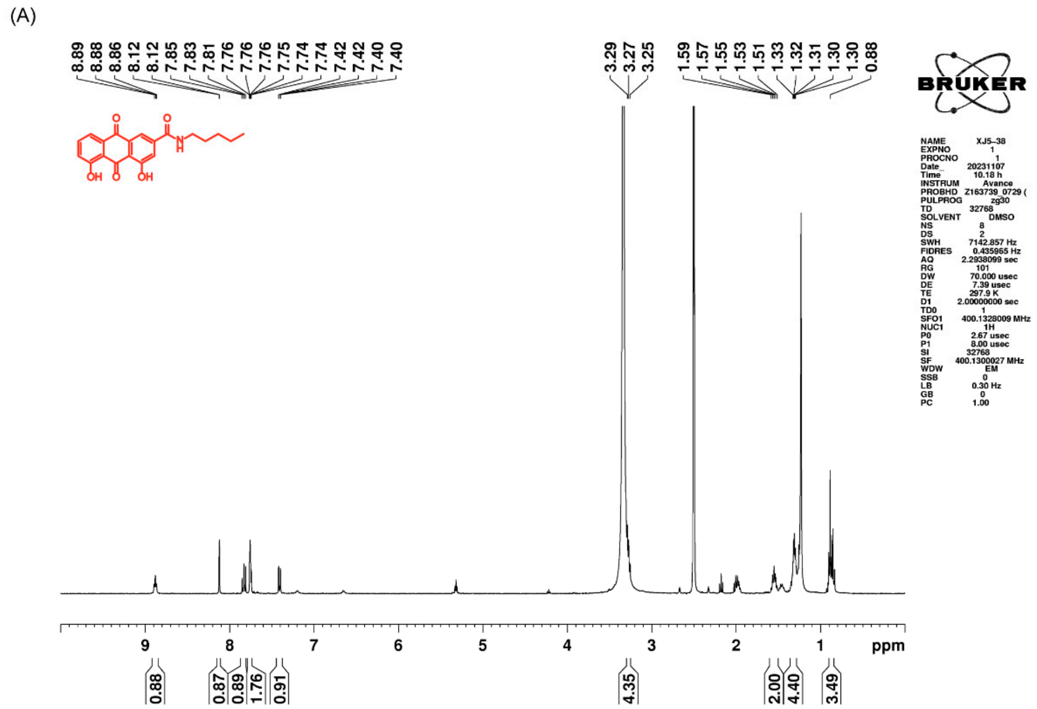

Figure S1 ^{1}H NMR (400 MHz, DMSO$_{d6}$) (A) spectrum of RH**1**.

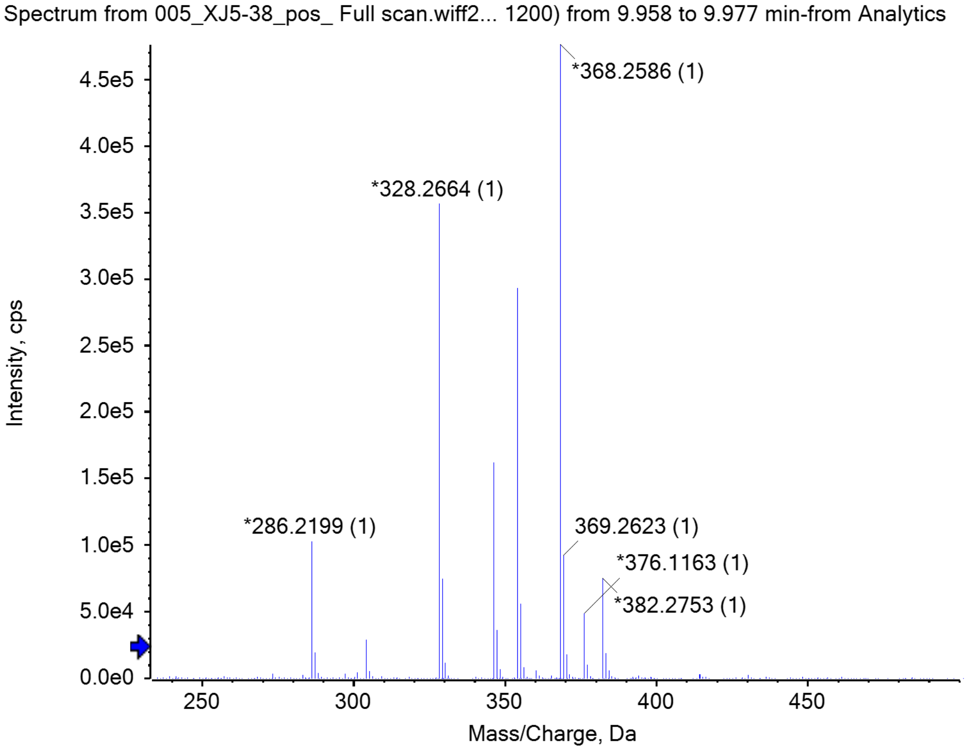

Figure S2 HR-ESI-MS spectrum of RH**1**.

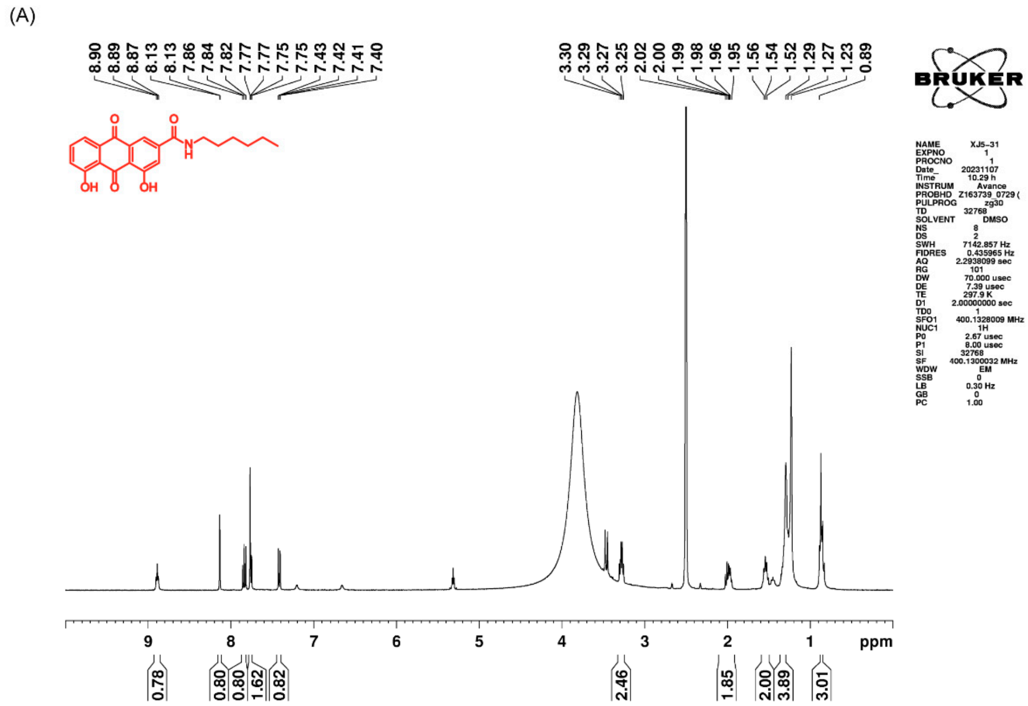

Figure S3 ^{1}H NMR (400 MHz, DMSO*d6*) (A) spectrum of RH**2**.

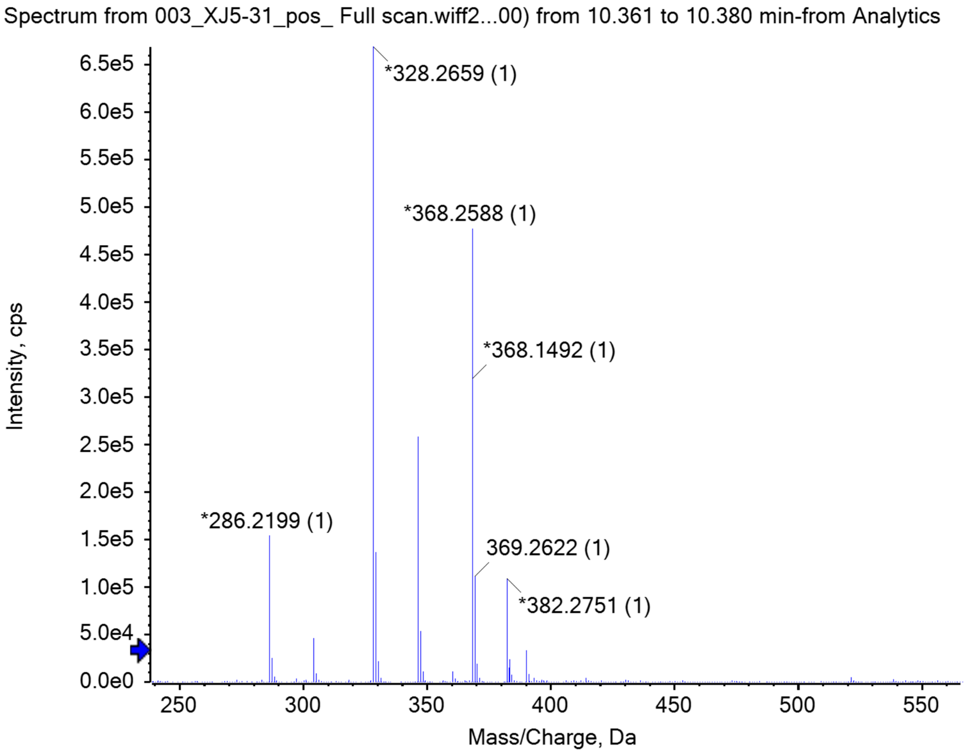

Figure S4 HR-ESI-MS spectrum of RH**2**.

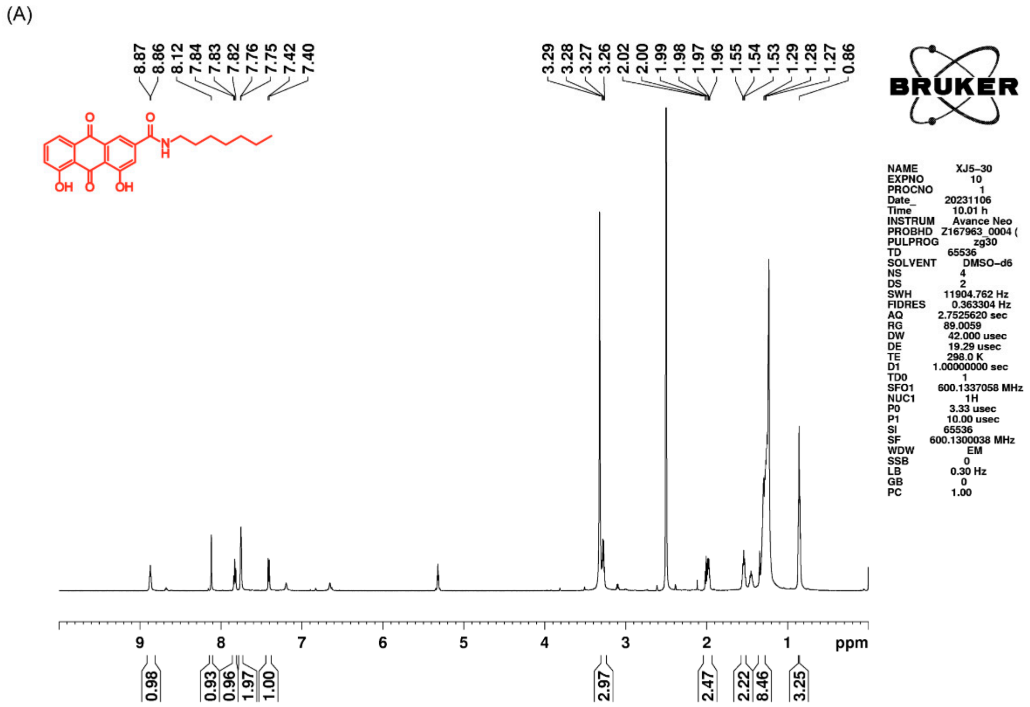

Figure S5 ^{1}H NMR (600 MHz, DMSO$_{d6}$) (A) spectrum of RH**3**.

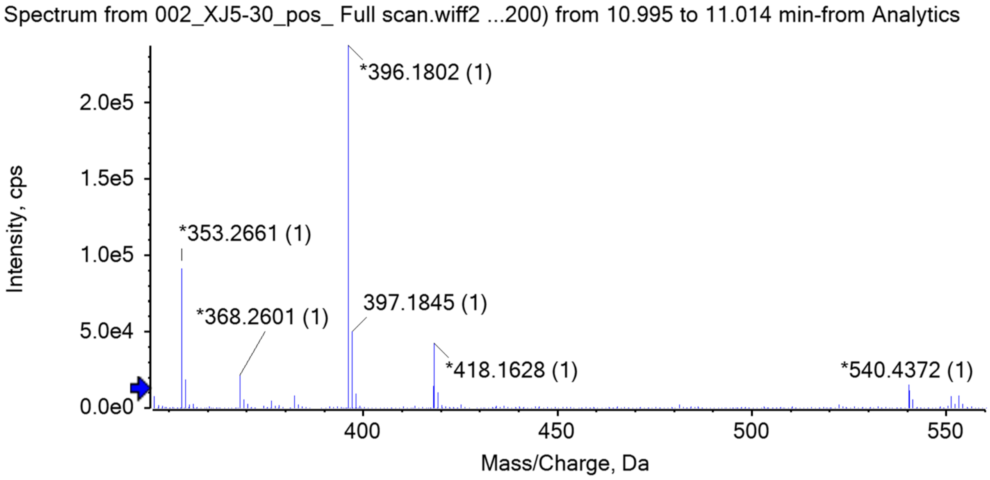

Figure S6 HR-ESI-MS spectrum of RH**3**.

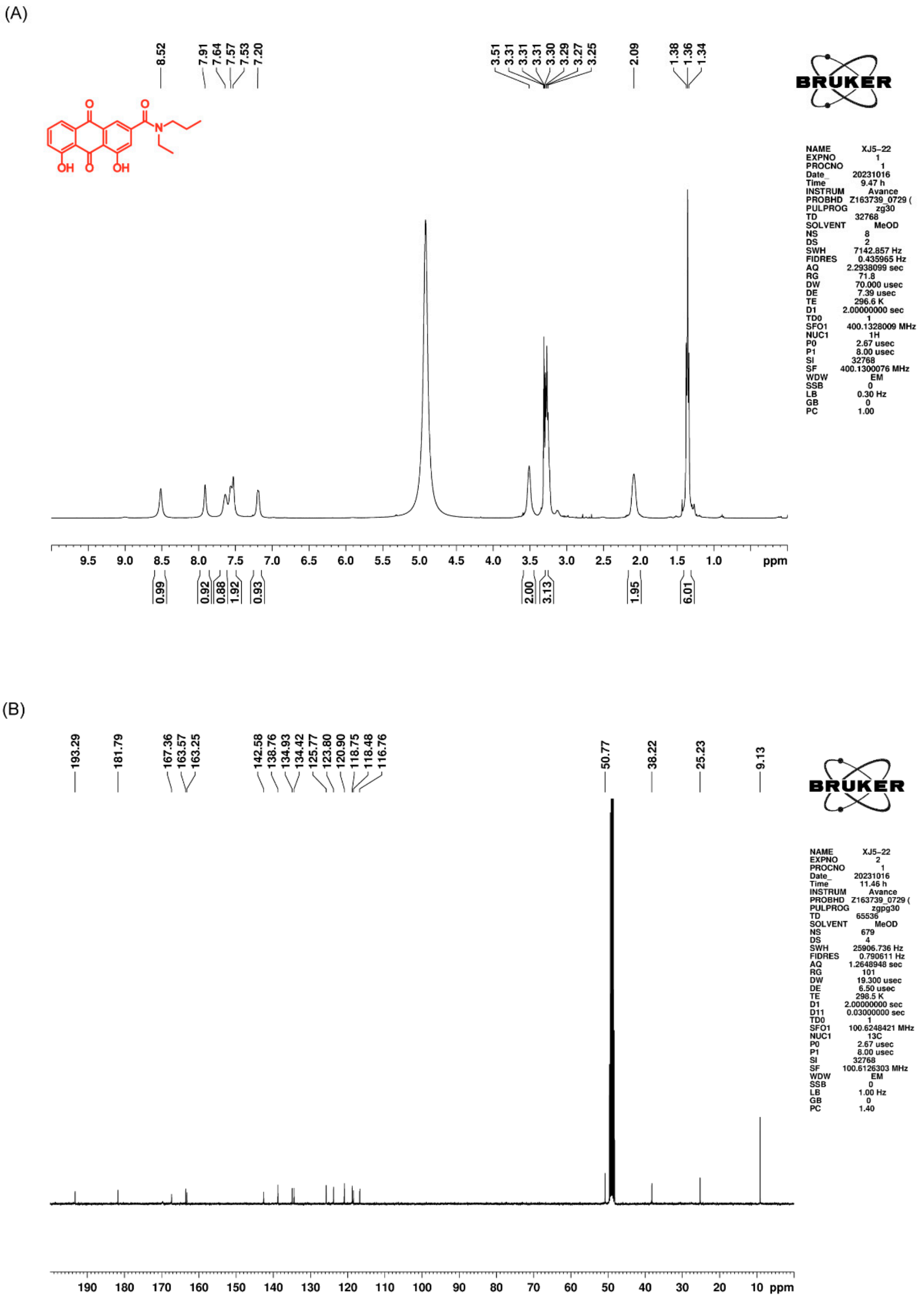

Figure S7 ^{1}H NMR (400 MHz, CD_3OD) (A) and ^{13}C NMR (100 MHz, CD_3OD) (B) spectrum of RH**4**.

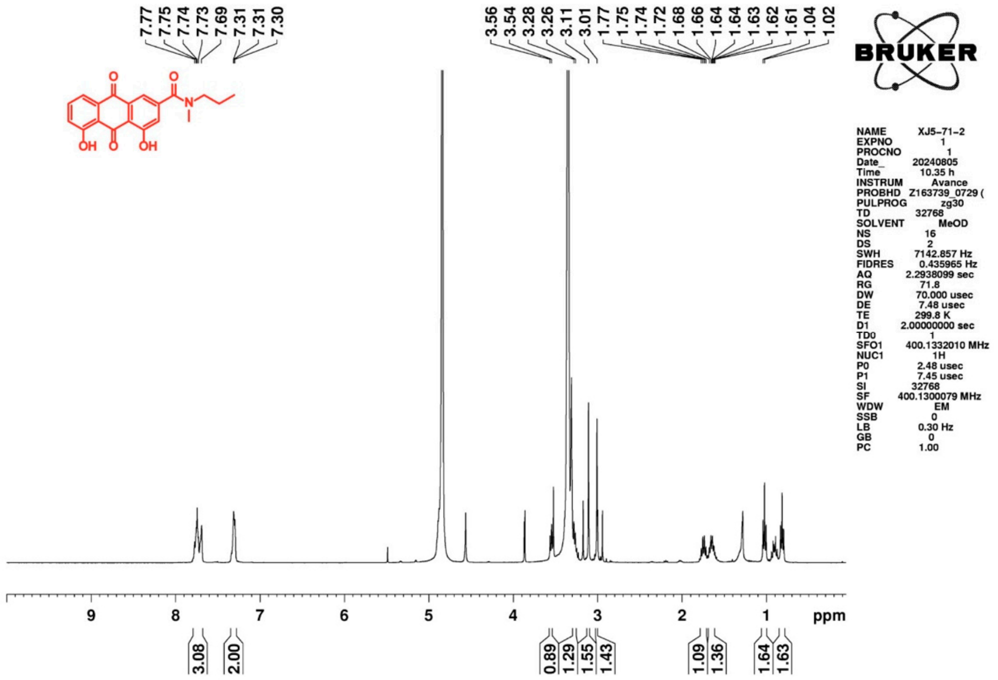

(B)

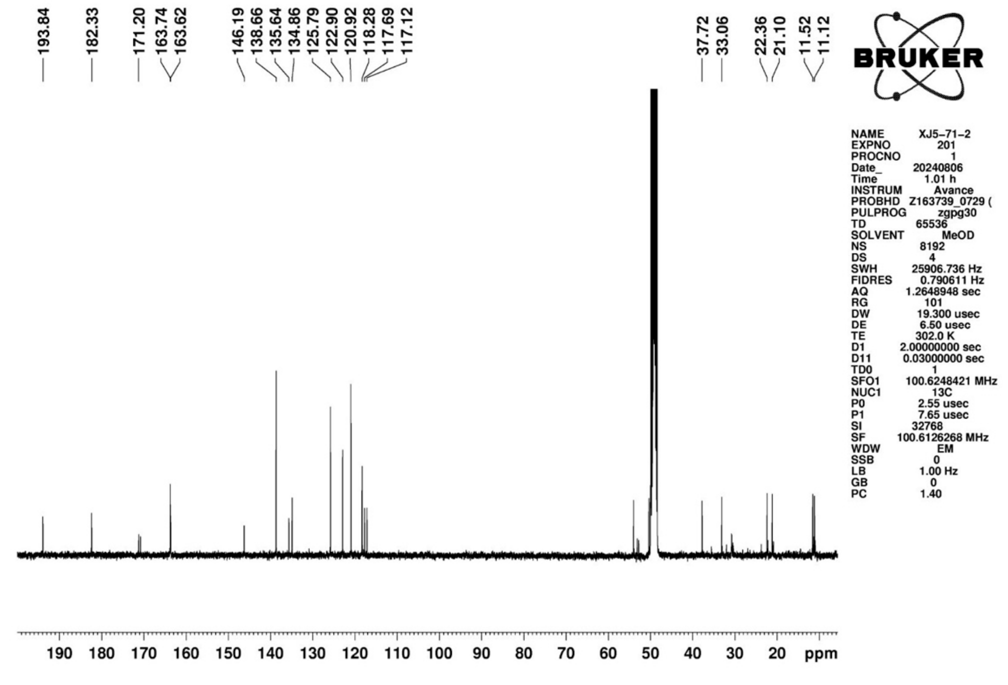

Figure S8 ^{1}H NMR (400 MHz, CD_3OD) (A) and ^{13}C NMR (100 MHz, CD_3OD) (B) spectrum of RH**5**.

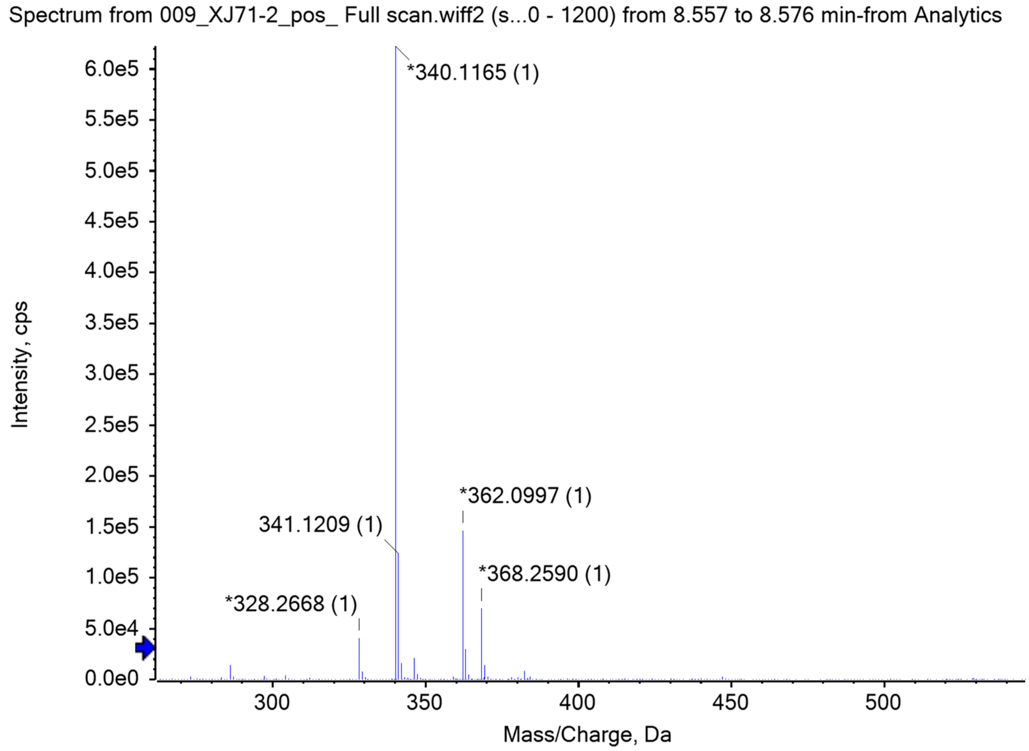

Figure S9 HR-ESI-MS spectrum of RH**5**.

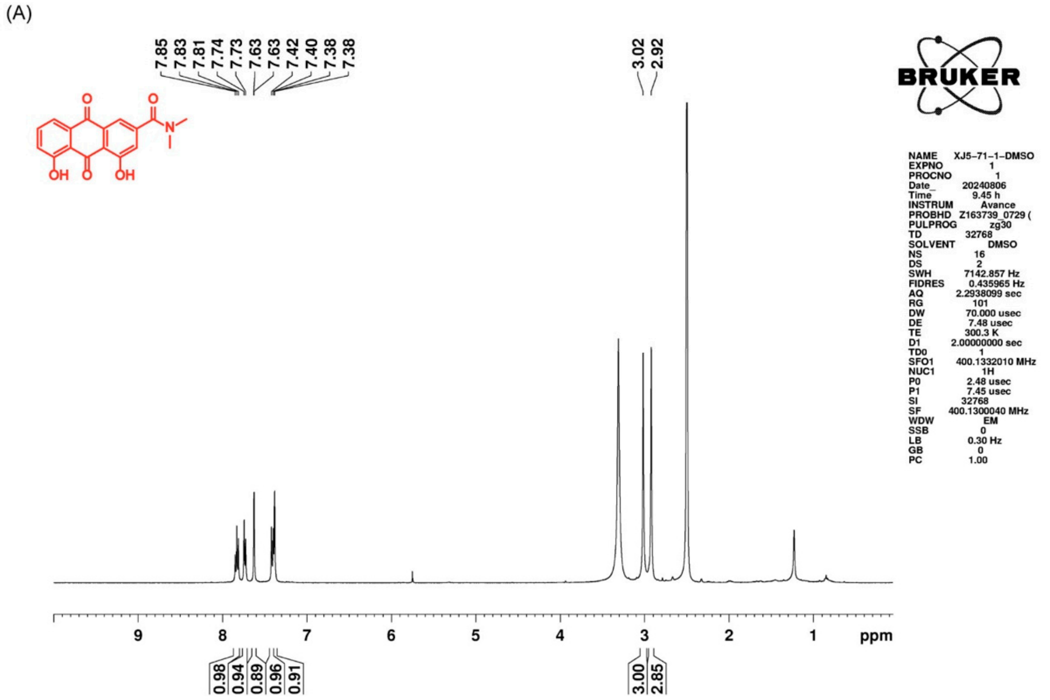

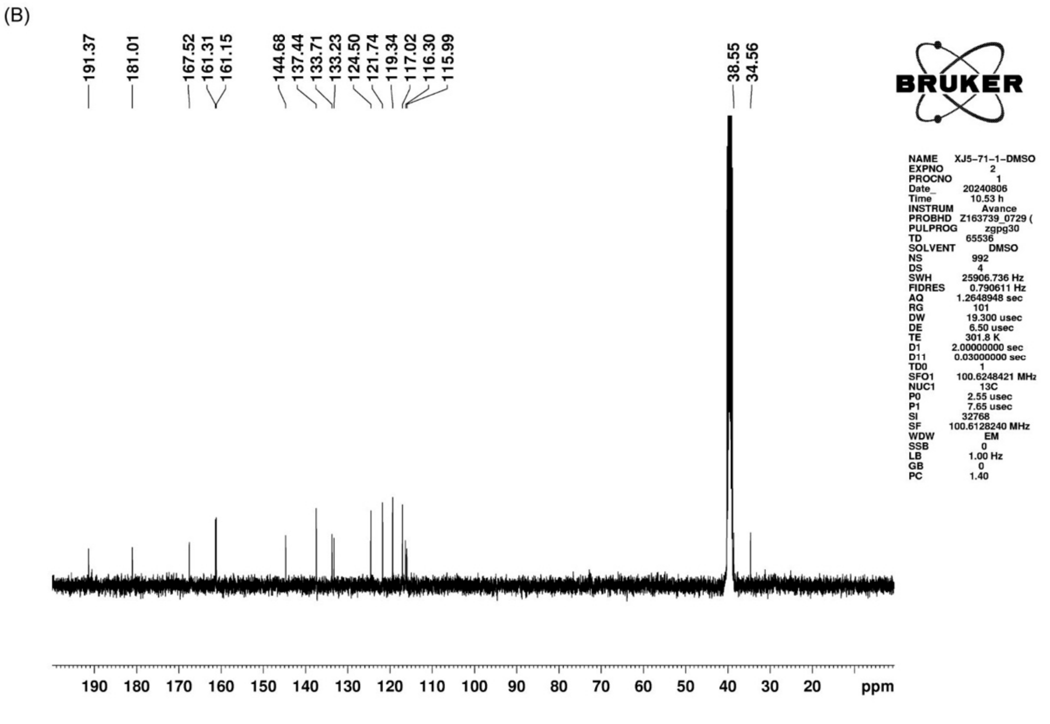

Figure S10 ^{1}H NMR (400 MHz, DMSO*d6*) (A) and ^{13}C NMR (100 MHz, DMSO*d6*) (B) spectrum of RH**6**.

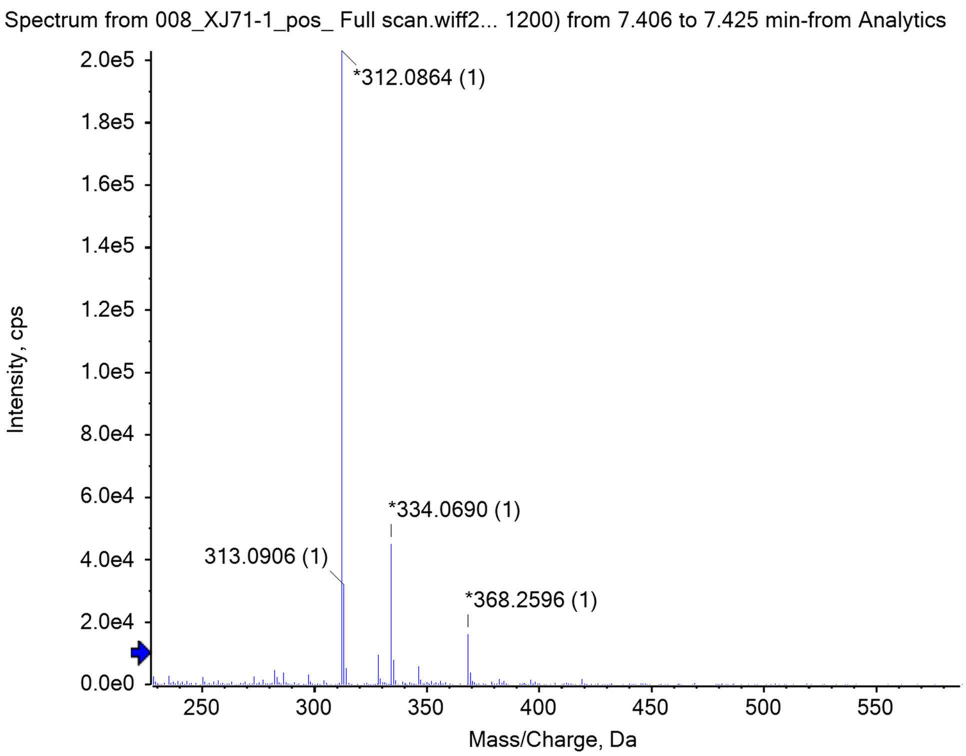

Figure S11 HR-ESI-MS spectrum of RH**6**.

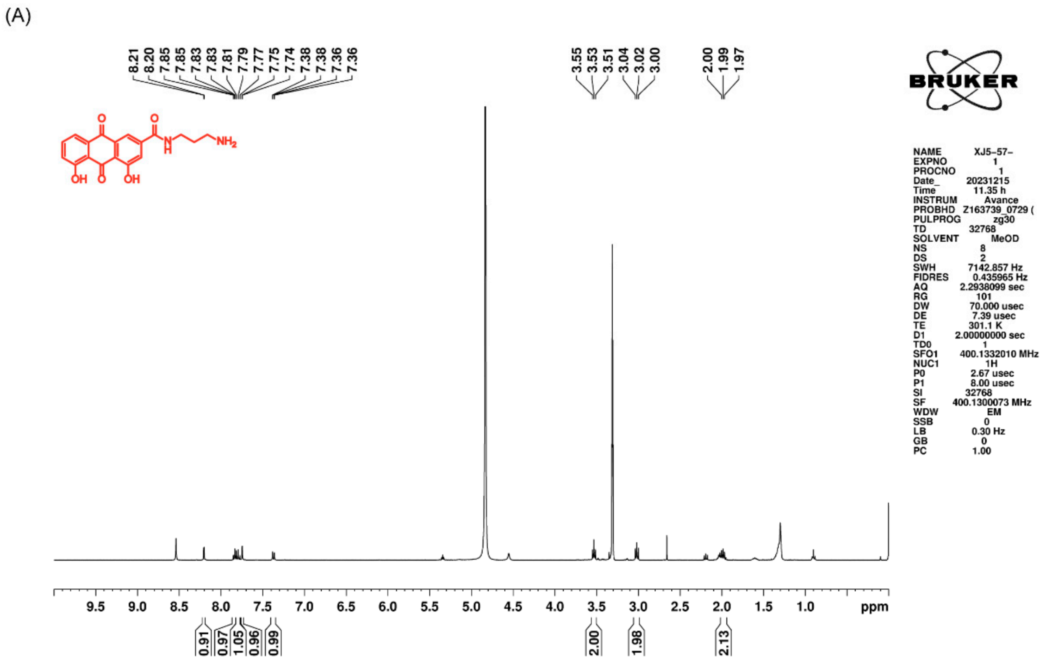

Figure S12 ^{1}H NMR (400 MHz, CD_3OD) (A) spectrum of RH**7**.

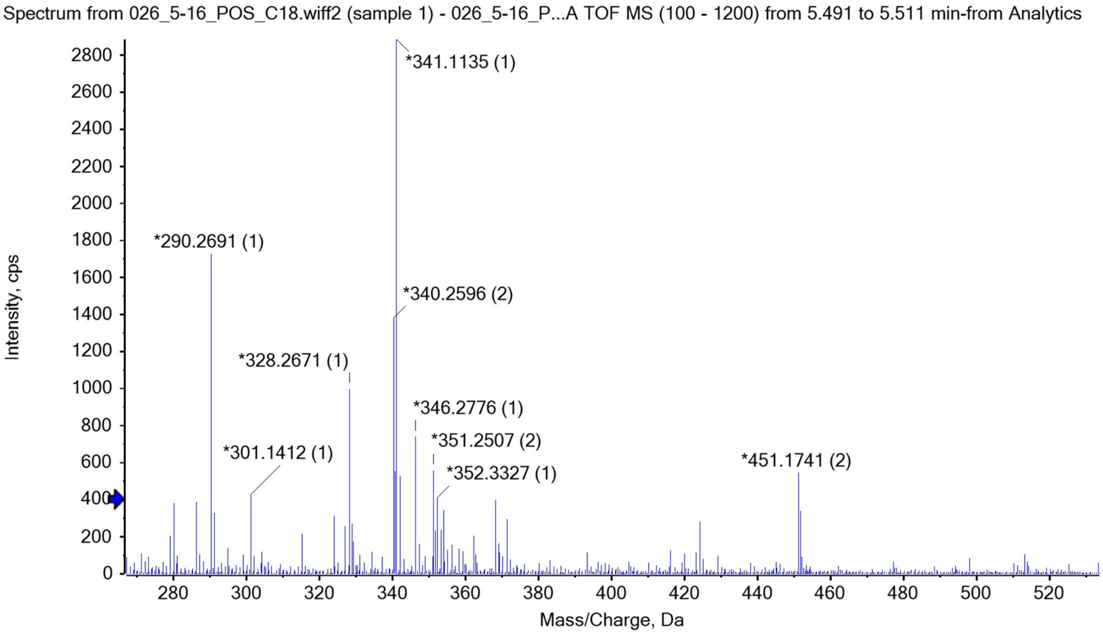

Figure S13 HR-ESI-MS spectrum of RH**7**.

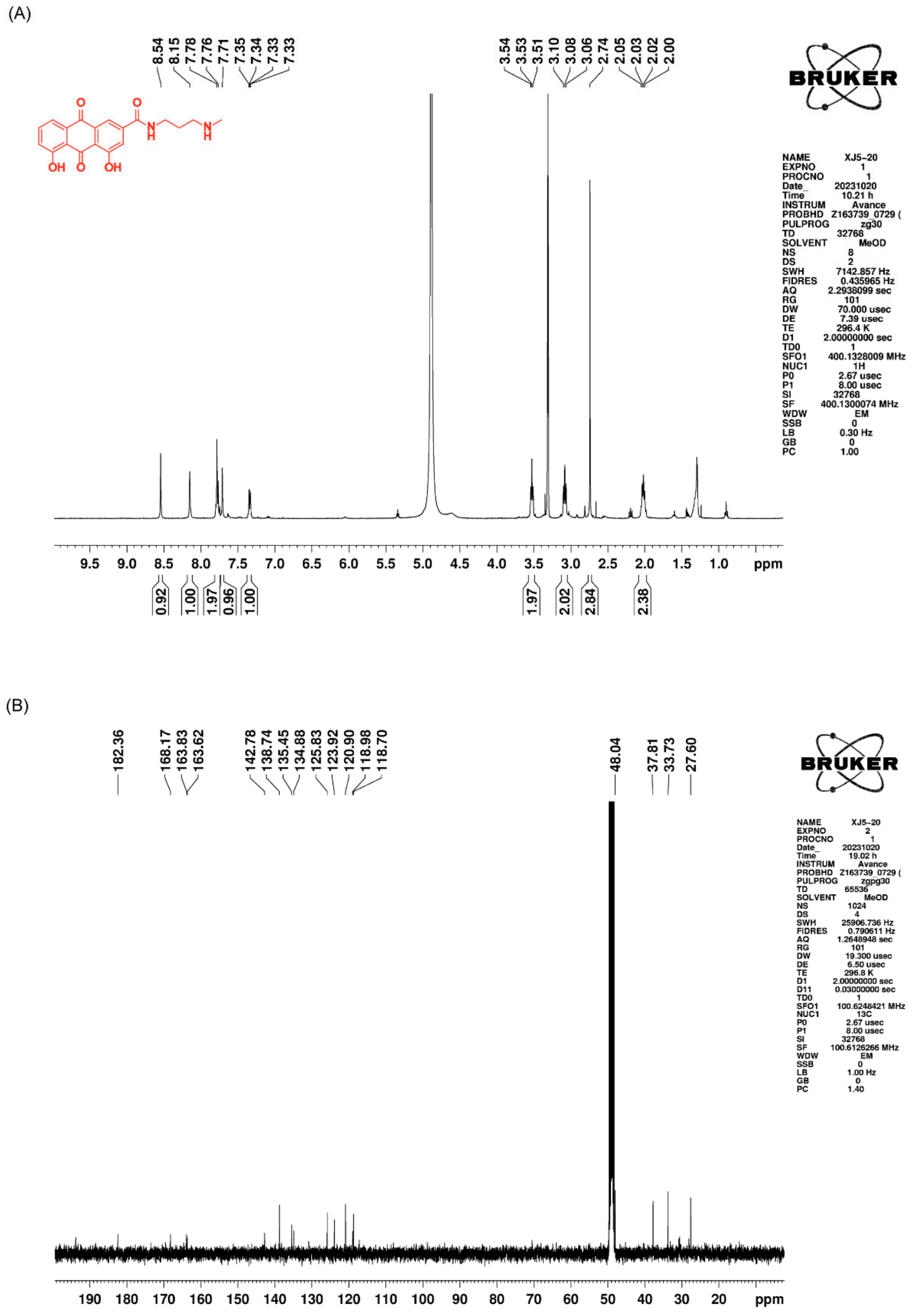

Figure S14 ^{1}H NMR (400 MHz, CD_3OD) (A) and ^{13}C NMR (100 MHz, CD_3OD) (B) spectrum of RH**8**.

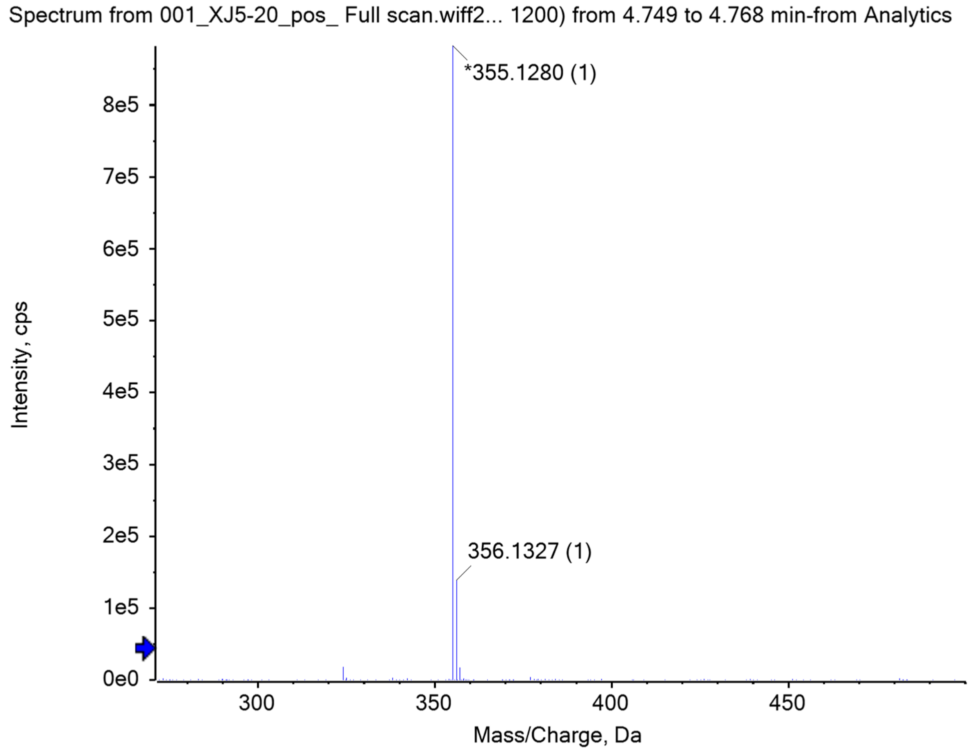

Figure S15 HR-ESI-MS spectrum of RH**8**.

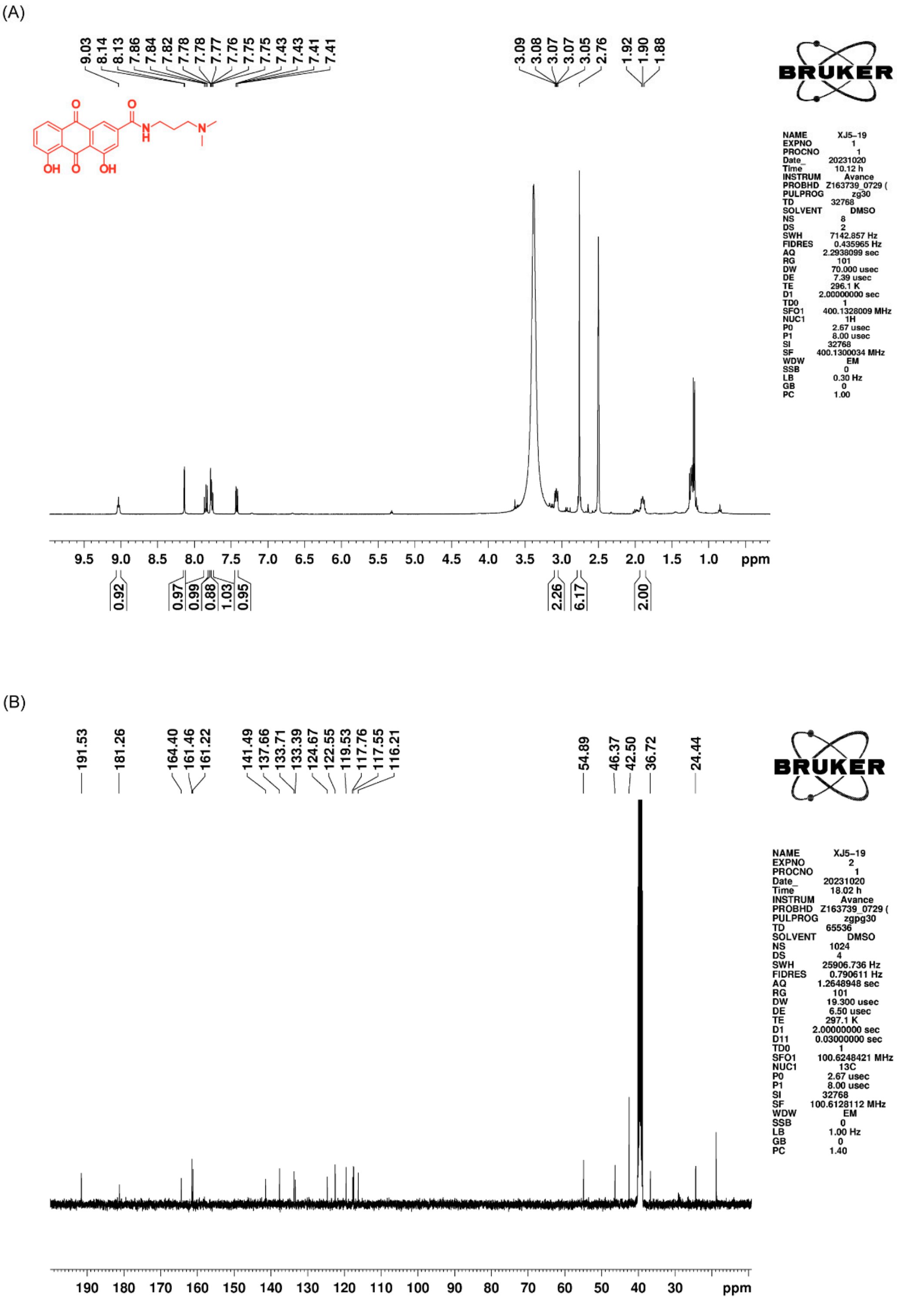

Figure S16 ^{1}H NMR (400 MHz, DMSO$_{d6}$) (A) and ^{13}C NMR (100 MHz, DMSO$_{d6}$) (B) spectrum of RH**9**.

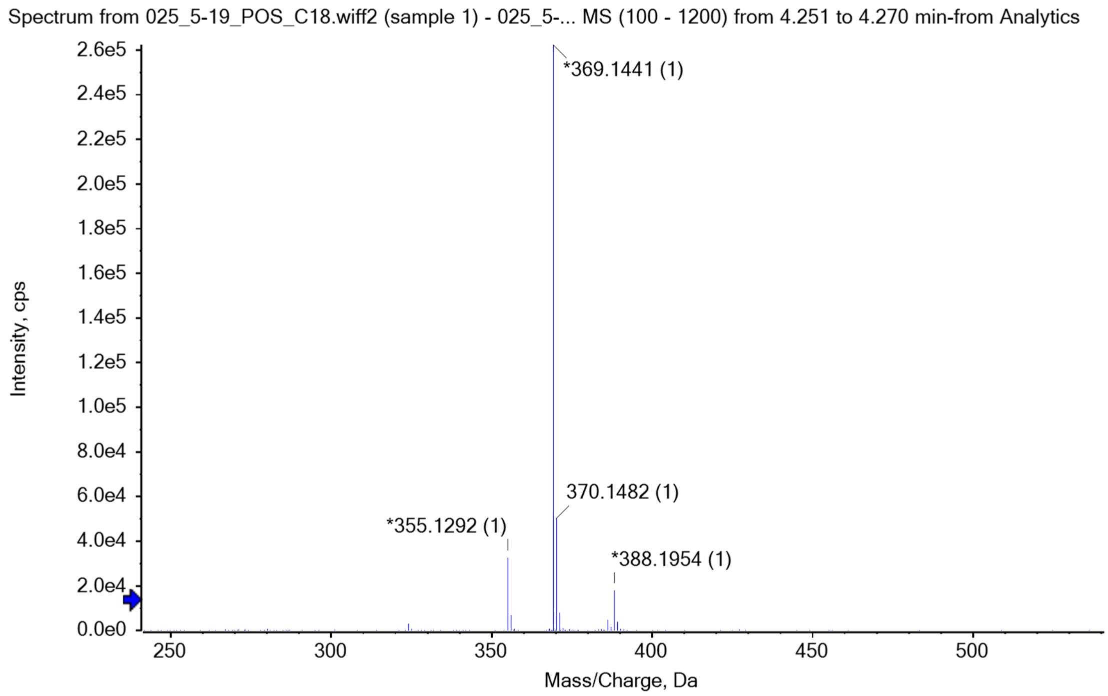

Figure S17 HR-ESI-MS spectrum of RH**9**.

(A)

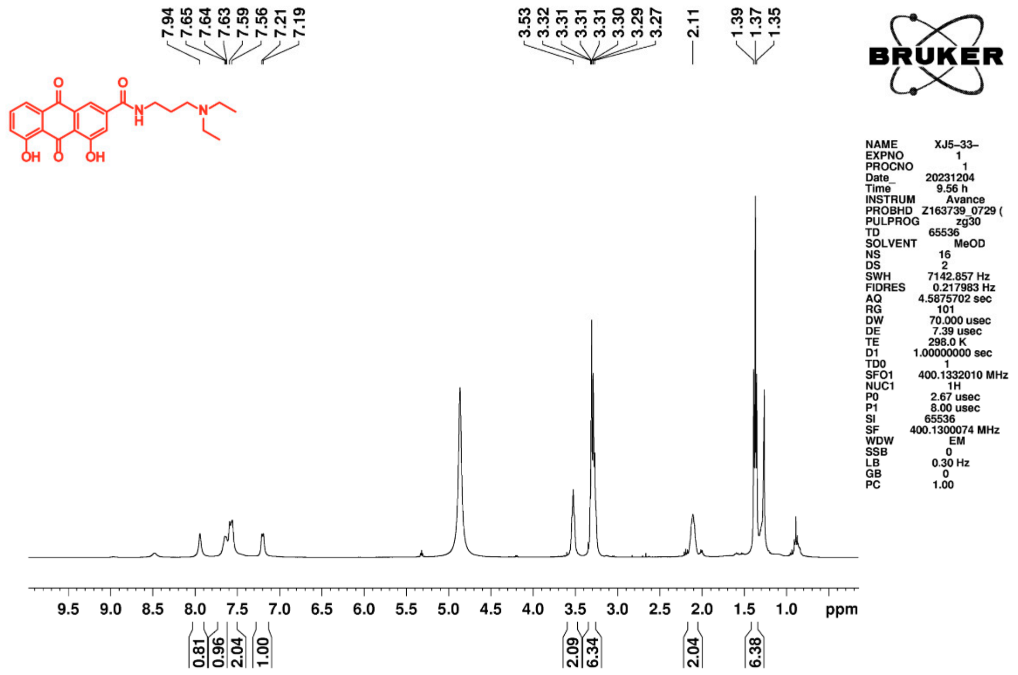

(B)

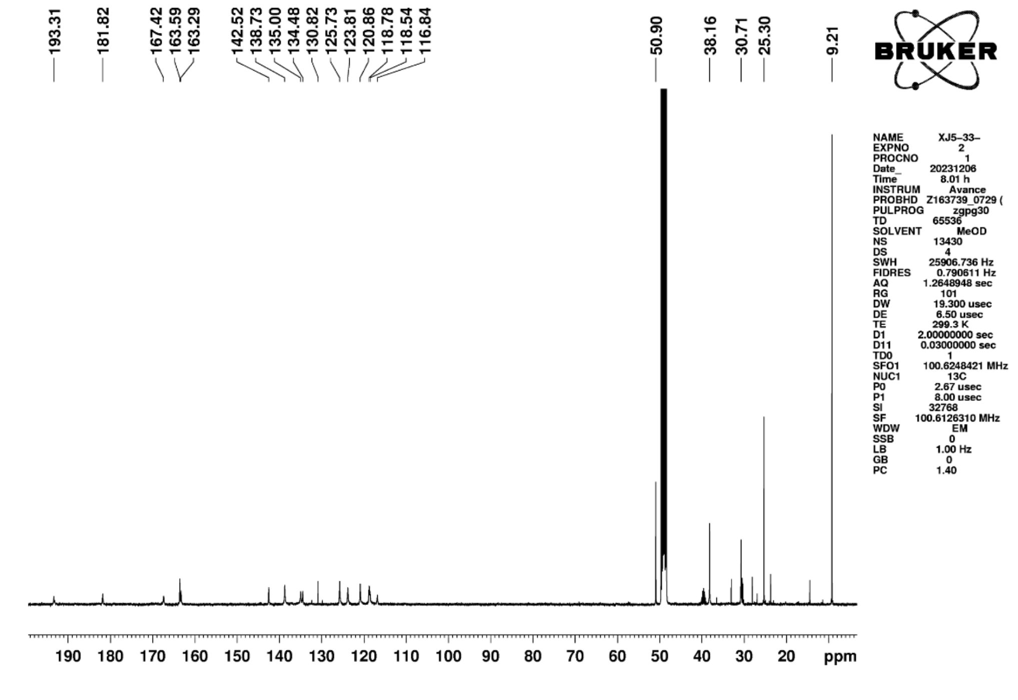

Figure S18 ^{1}H NMR (400 MHz, CD_3OD) (A) and ^{13}C NMR (100 MHz, CD_3OD) (B) spectrum of RH**10**.

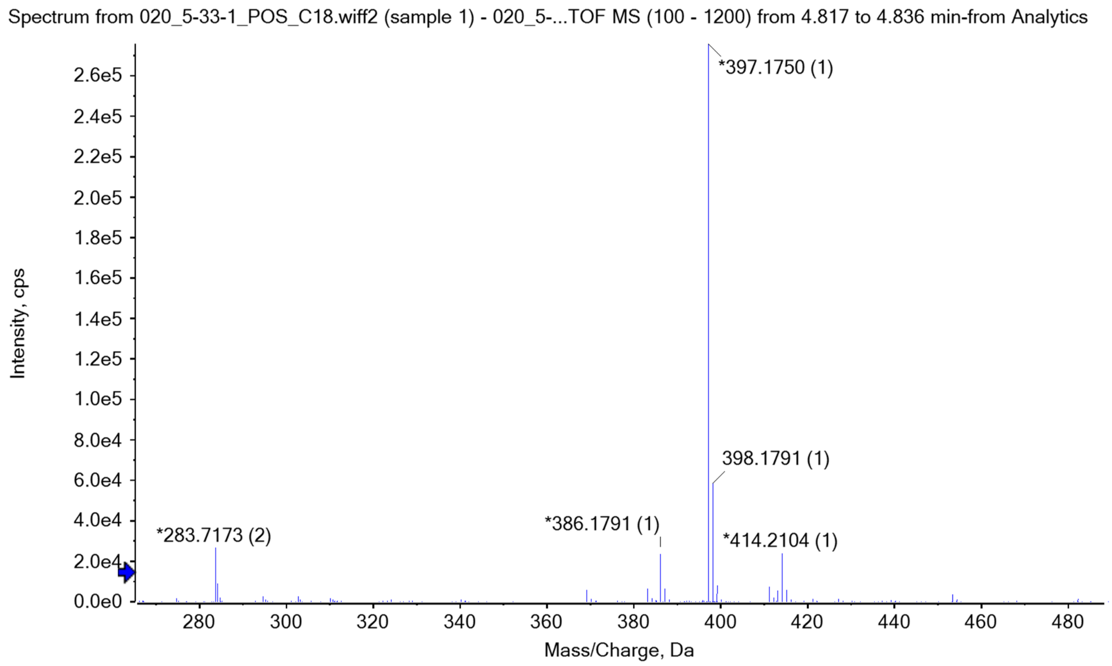

Figure S19 HR-ESI-MS spectrum of RH**10**.

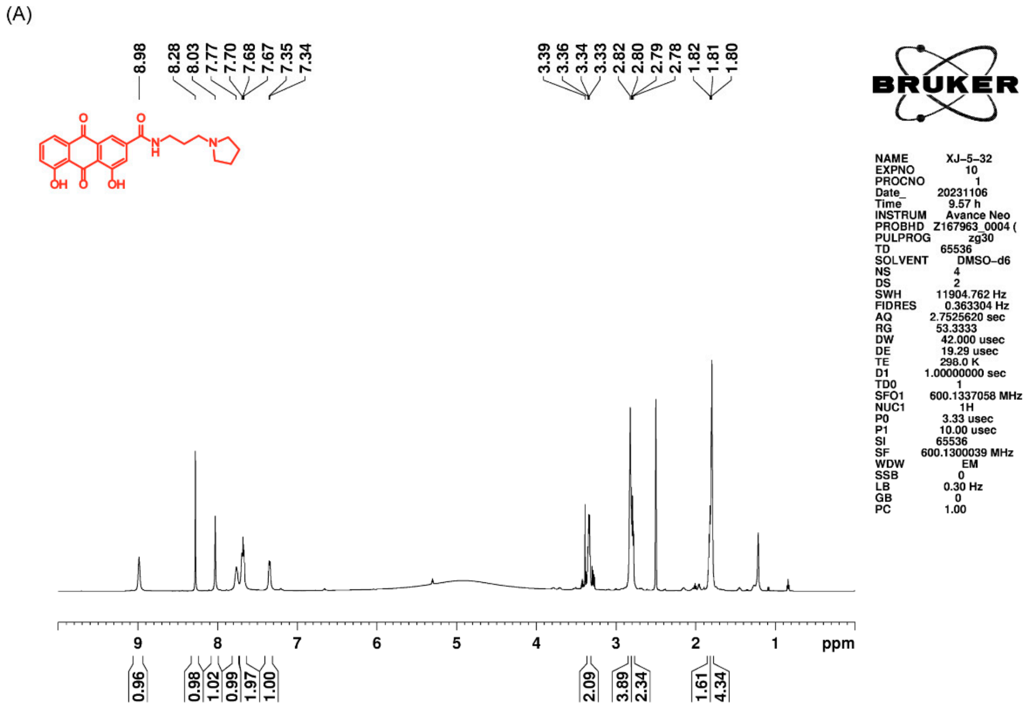

Figure S20 ^{1}H NMR (600 MHz, DMSO$_{d6}$) (A) spectrum of RH**11**.

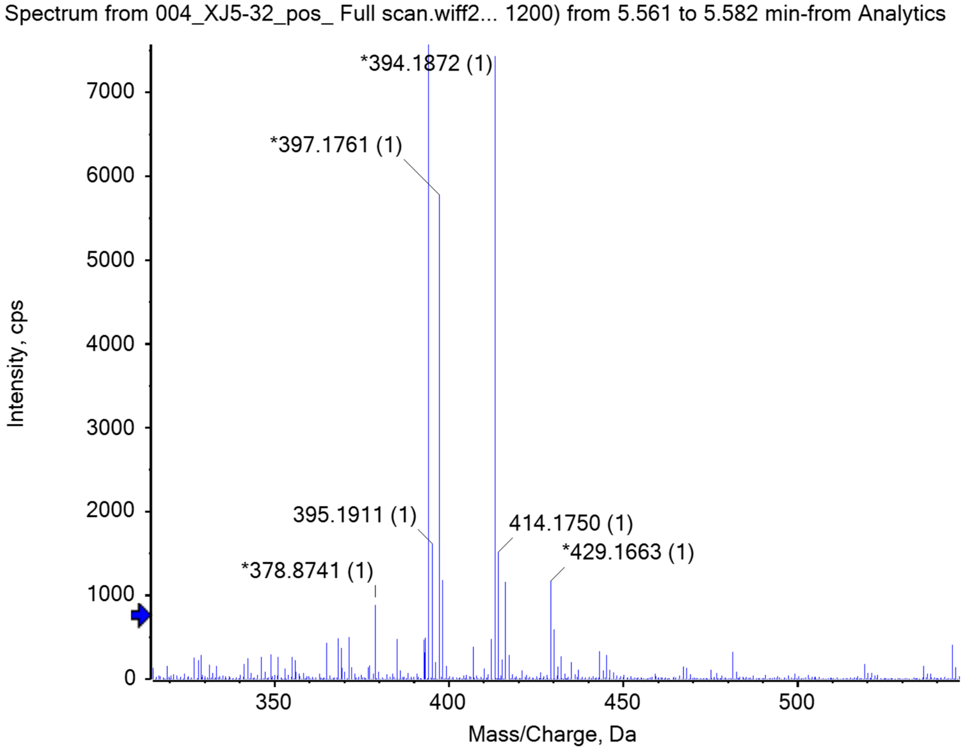

Figure S21 HR-ESI-MS spectrum of RH**11**.

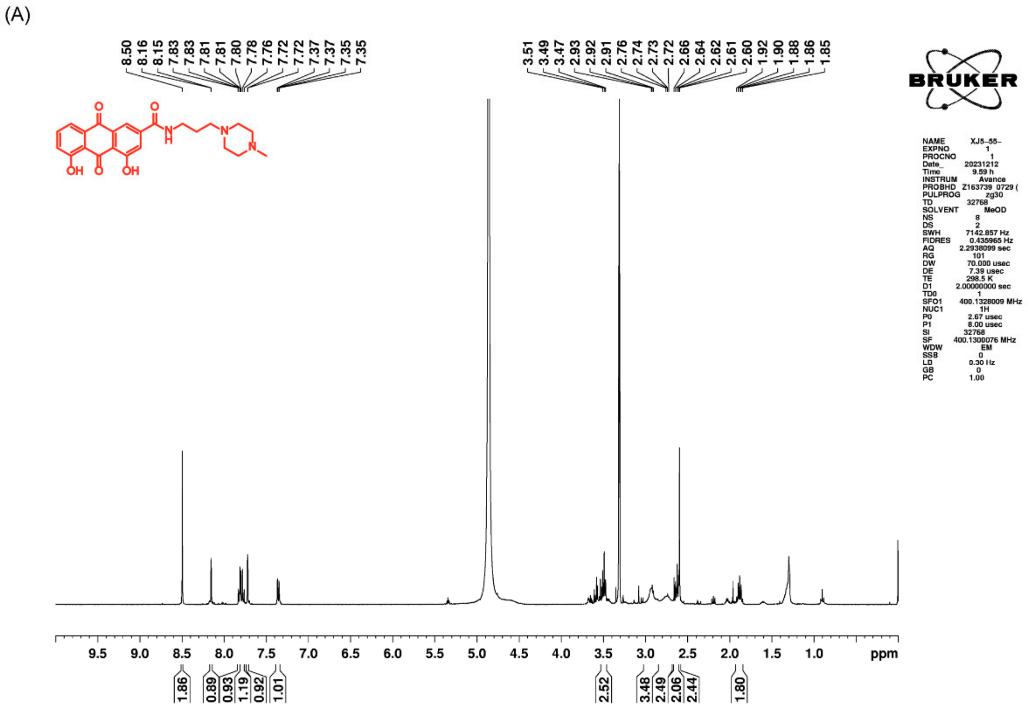

Figure S22 ^{1}H NMR (400 MHz, CD_3OD) (A) spectrum of RH**12**.

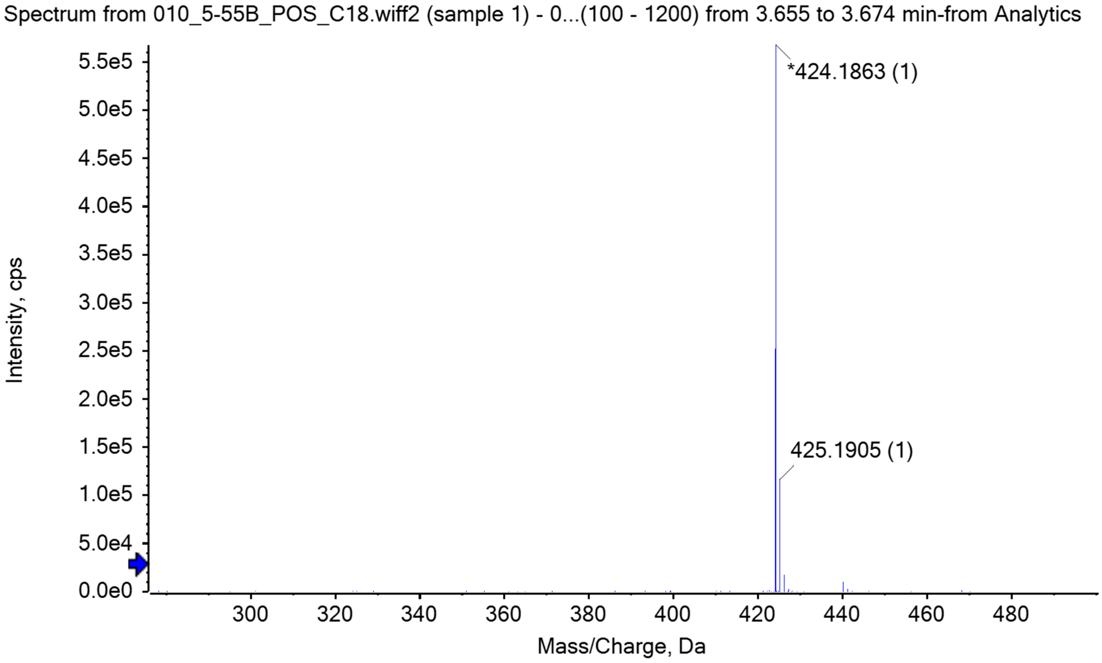

Figure S23 HR-ESI-MS spectrum of RH**12**.

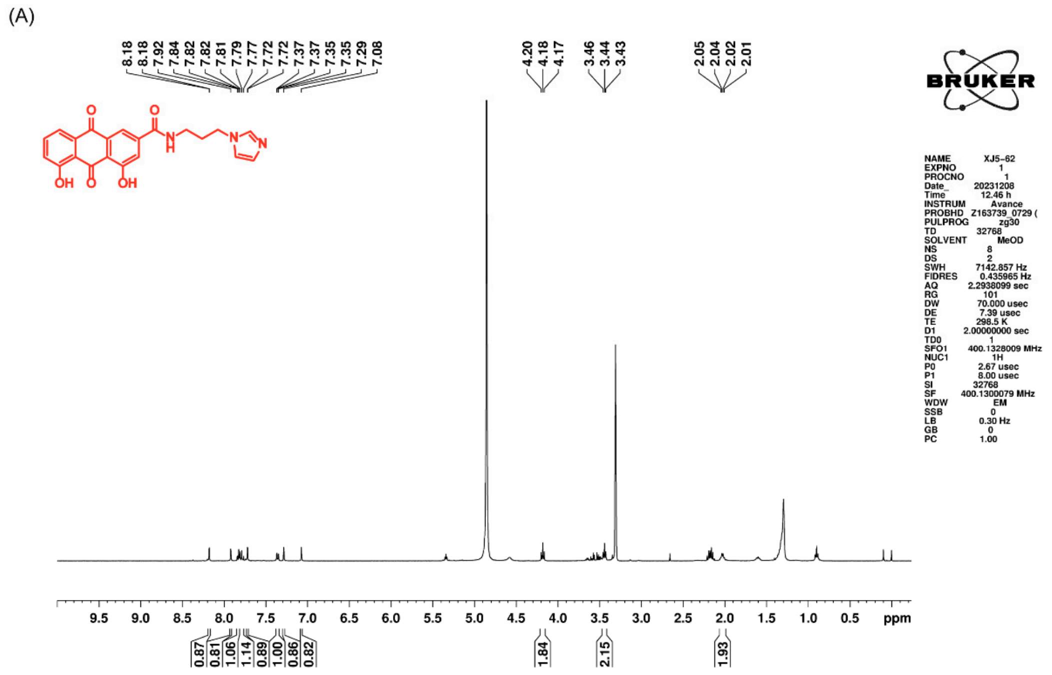

Figure S24 ^{1}H NMR (400 MHz, CD_3OD) (A) spectrum of RH**13**.

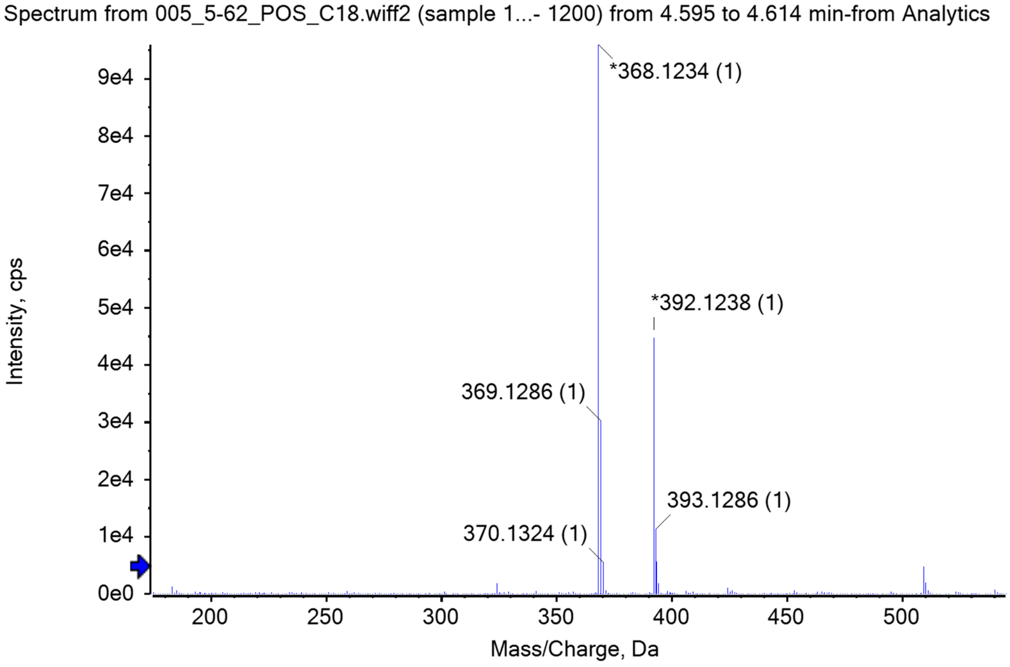

Figure S25 HR-ESI-MS spectrum of RH**13**.

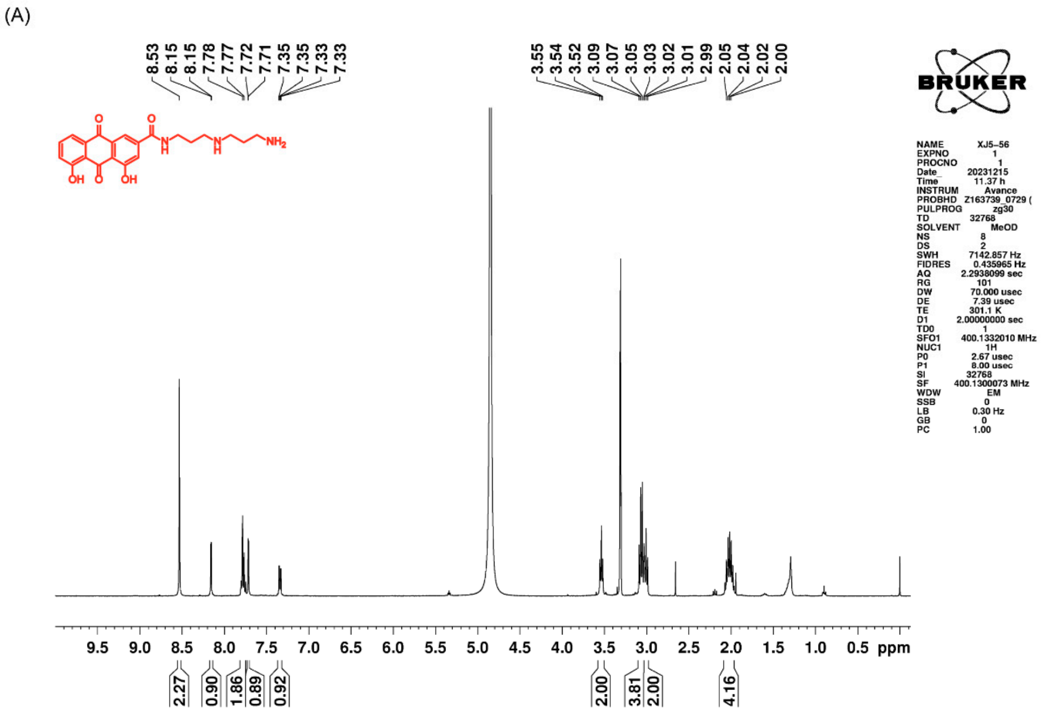

Figure S26 ^{1}H NMR (400 MHz, CD_3OD) (A) spectrum of RH**14**.

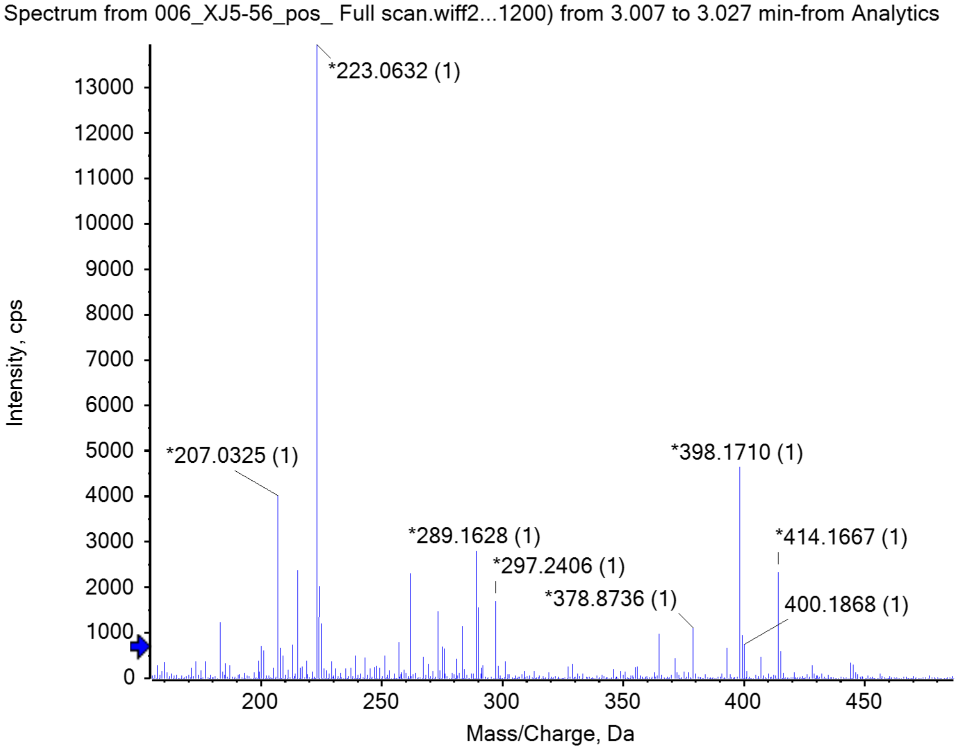

Figure S27 HR-ESI-MS spectrum of RH**14**.

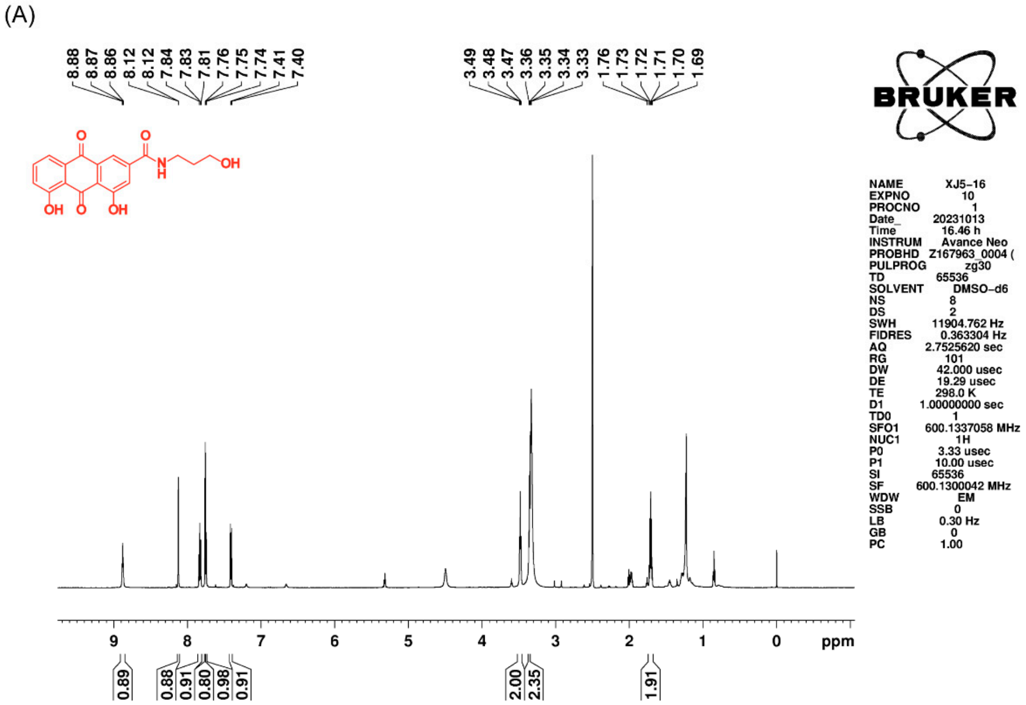

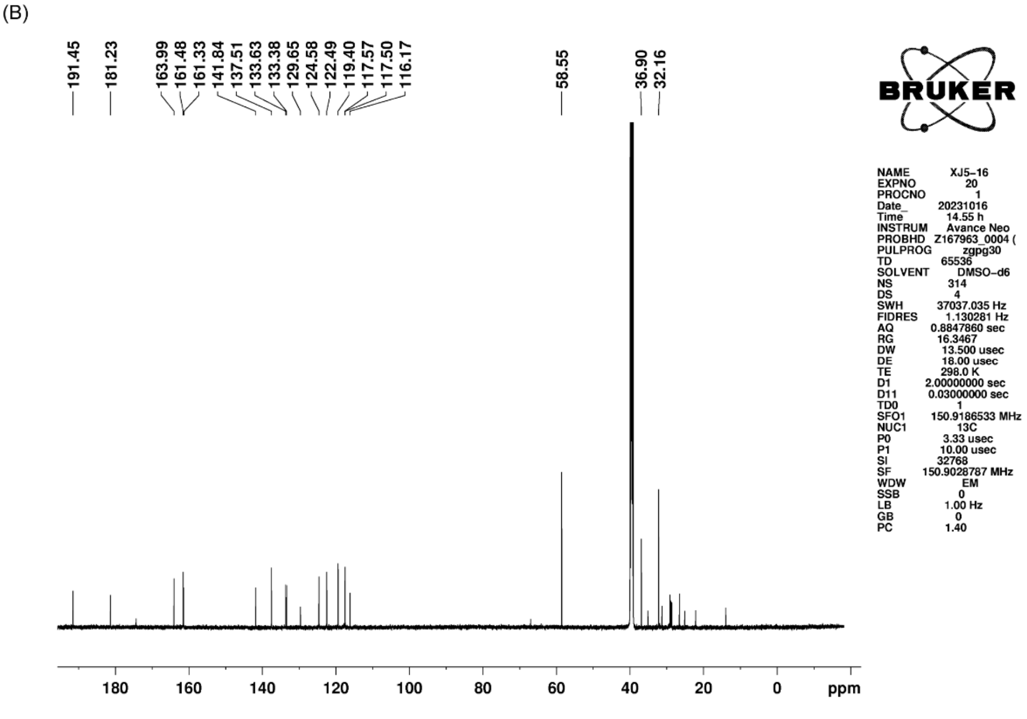

Figure S28 ^{1}H NMR (600 MHz, DMSO$_{d6}$) (A) and ^{13}C NMR (150 MHz, DMSO$_{d6}$) (B) spectrum of RH**15**.

(A)

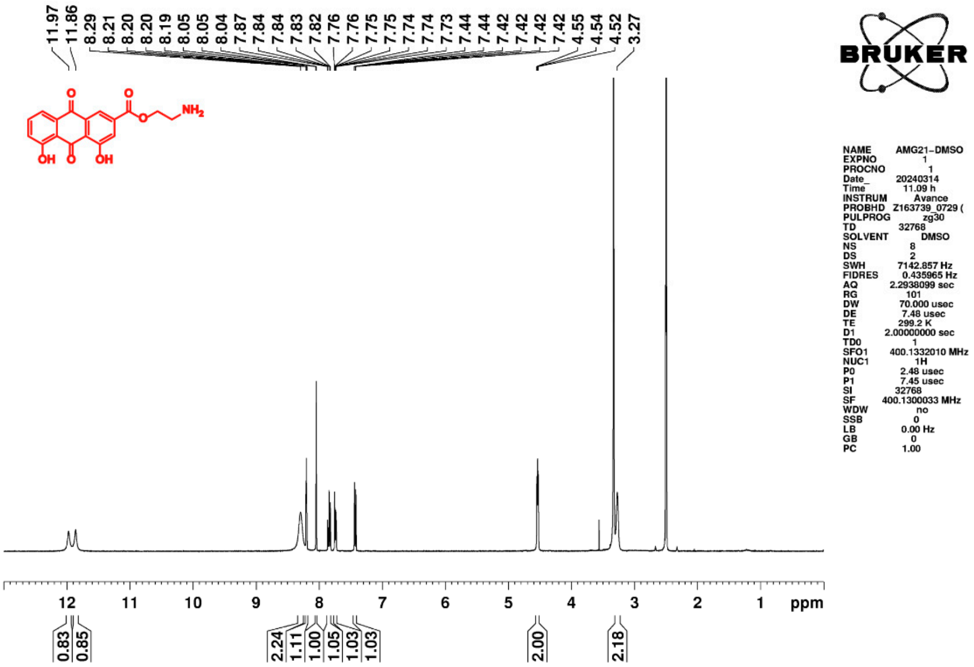

(B)

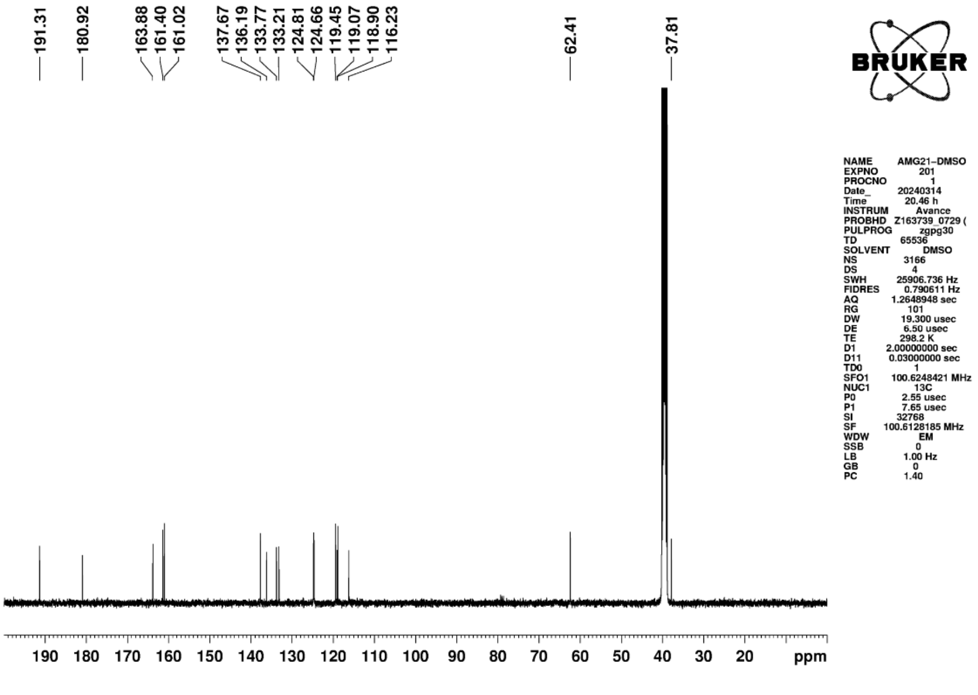

Figure S29 ^{1}H NMR (400 MHz, DMSO*d6*) (A) and ^{13}C NMR (100 MHz, DMSO*d6*) (B) spectrum of RH**17**.

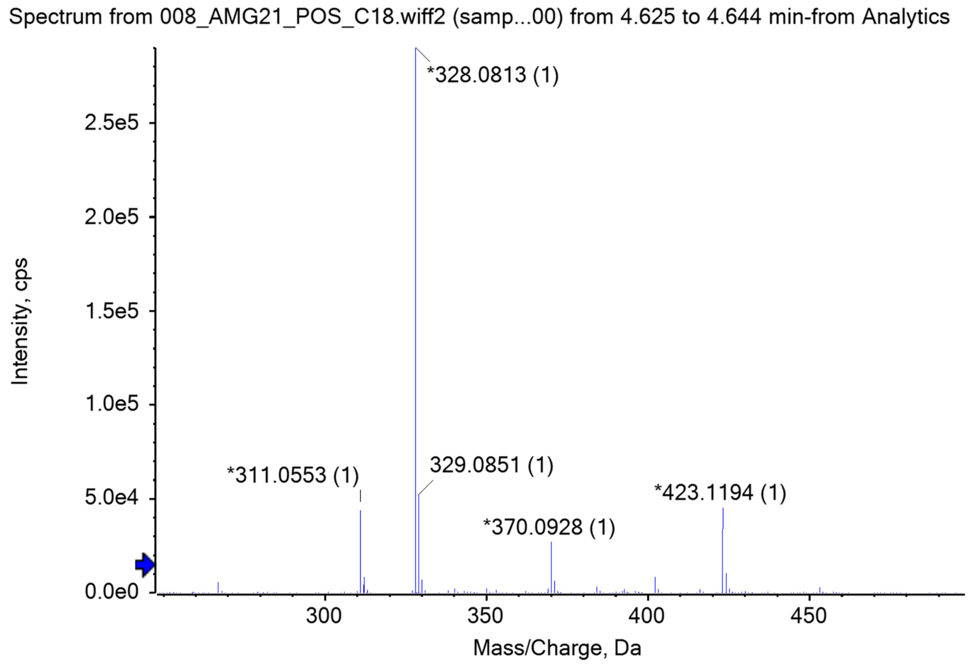

Figure S30 HR-ESI-MS spectrum of RH**17**.

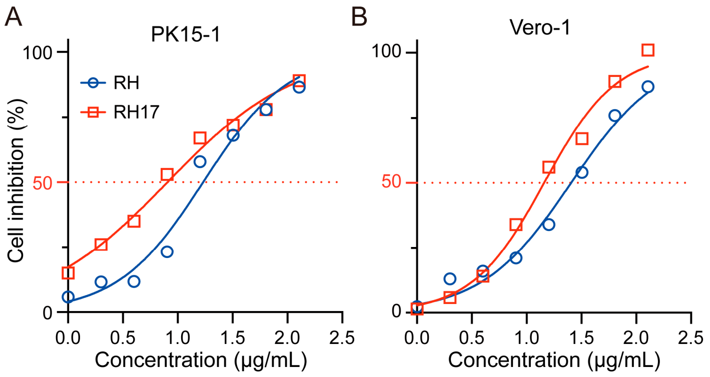

Figure S31 Cytotoxicity of RH and RH17. The IC_{50} values of RH against PK15-1 and Vero-1 cell lines were 17.03 and 25.45 μg/mL, respectively. The IC_{50} values of RH17 against PK15-1 and Vero-1 cell lines were 8.06 and 14.44 μg/mL, respectively.

Table S1 MIC and MBC values of RH and RH17 against *S. aureus* isolates (n = 40).

No.	Strains	Description	RH (μg/mL)		RH17 (μg/mL)	
			MIC	MBC	MIC	MBC
1	*S. aureus* ATCC 29213	MSSA	32	128	8	16
2	*S. aureus* G1	MSSA	32	128	8	16
3	*S. aureus* G2	MSSA	32	64	8	16
4	*S. aureus* G3	MSSA	128	> 128	8	32
5	*S. aureus* G4	MSSA	32	128	8	16
6	*S. aureus* G5	MSSA	64	128	16	32
7	*S. aureus* SHY37-1	MSSA	32	128	8	16
8	*S. aureus* S2AY8	MSSA	32	128	8	16
9	*S. aureus* SEY16-1	MSSA	64	> 128	8	32
10	*S. aureus* S1AH053	MSSA	32	64	8	16
11	*S. aureus* SAY42	MSSA	64	128	16	64
12	*S. aureus* S1AJ062	MSSA	32	128	8	16
13	*S. aureus* S2AY23	MSSA	32	128	8	16
14	*S. aureus* S2AY11	MSSA	64	128	8	32
15	*S. aureus* G7	MSSA	32	64	8	16
16	*S. aureus* A187	MSSA	32	128	8	16
17	*S. aureus* GD705	MSSA	32	128	8	16
18	*S. aureus* GD1677	MSSA	64	> 128	8	16
19	*S. aureus* 43P8	MSSA	32	128	8	16
20	*S. aureus* 88088-2	MSSA	32	64	8	16
21	*S. aureus* BA01611	MRSA	64	128	8	16
22	*S. aureus* TSAR03	MRSA	32	128	8	16
23	*S. aureus* TSAR04	MRSA	32	128	8	16

24	*S. aureus* TSAR07	MRSA	64	128	16	32
25	*S. aureus* TSAR08	MRSA	32	128	8	16
26	*S. aureus* TSAR01	MRSA	32	128	16	64
27	*S. aureus* SAV1158	MRSA	64	128	8	32
28	*S. aureus* SAV1150	MRSA	32	128	16	64
29	*S. aureus* SAV1146	MRSA	32	64	8	16
30	*S. aureus* SAV1109	MRSA	64	128	16	64
31	*S. aureus* DL44	MRSA	64	128	8	16
32	*S. aureus* DY82	MRSA	32	64	8	16
33	*S. aureus* QD-T9	MRSA	64	> 128	16	32
34	*S. aureus* YH022	MRSA	64	> 128	8	32
35	*S. aureus* DG35	MRSA	32	128	16	64
36	*S. aureus* DG38	MRSA	32	128	8	16
37	*S. aureus* DG10	MRSA	64	> 128	16	32
38	*S. aureus* DG24	MRSA	32	128	8	16
39	*S. aureus* DG12	MRSA	32	128	16	64
40	*S. aureus* YN502	MRSA	64	128	8	16
